# Outer membrane vesicles secreted by avian pathogenic *Escherichia coli* promote intracellular survival in macrophages and systemic infection by regulating mitophagy through the MSTRG.11745.1/gga-miR-15b-5p/*TNFAIP3* axis

**DOI:** 10.1016/j.psj.2026.107755

**Published:** 2026-09-13

**Authors:** Jiayin Gao, Zhe Li, Tongtong Cui, Ying Shao, Jiumeng Sun, Zhenyu Wang, Xiangjun Song, Zhengyang Cheng, Jian Tu

**Affiliations:** a Anhui Province Key Laboratory of Veterinary Pathobiology and Disease Control, College of Veterinary Medicine, Anhui Agricultural University, Hefei, 230036, China; b Anhui Province Engineering Labora tory for Animal Food Quality and Bio-Safety, College of Veterinary Medicine, Anhui Agricultural University, Hefei, 230036, China; c Joint Research Center for Food Nutrition and Health of IHM, Hefei, 230036, China

**Keywords:** Outer membrane vesicles of avian pathogenic *Escherichia coli*, Mitochondrial damage, Mitophagy, ceRNA, Immune evasion

## Abstract

Avian pathogenic *Escherichia coli* (**APEC**) is a major extraintestinal pathogen responsible for severe systemic infections in poultry. It evades the immune system and establishes persistent infection by hijacking macrophages as its primary site of intracellular survival. During the pathogenic process, outer membrane vesicles (**OMVs**) secreted by APEC serve as key carriers for the delivery of virulence factors to host cells. However, the specific mechanisms by which OMVs disrupt macrophage defence mechanisms and promote the intracellular survival of APEC have not yet been fully elucidated. We demonstrated that APEC OMVs disrupt the mitochondrial structure of chicken macrophages **(HD11**) and lead to the collapse of the mitochondrial membrane potential. This injury-induced mitophagy contributes to a reduction in stress-related ROS levels, thereby promoting the intracellular survival of APEC and systemic infection. Furthermore, intervention with a mitophagy inhibitor significantly reduced the extent of intracellular survival resulting from mitophagy (*P* < 0.01) as well as the bacterial load in chick tissues (trachea: *P* < 0.001; lung: *P* < 0.001; liver: *P* < 0.001; spleen: *P* < 0.01). Furthermore, We identified a novel lncRNA—MSTRG.11745.1—associated with mitophagy through transcriptomic screening, and upregulated MSTRG.11745.1 acts as a competitive endogenous RNA (**ceRNA**), binding to gga-miR-15b-5p to regulate the *TNFAIP3* expression, thereby promoting mitophagy and the intracellular survival of APEC. In summary, APEC-derived OMVs induce severe mitochondrial damage in host macrophages. They also activate the MSTRG.11745.1/gga-miR-15b-5p/*TNFAIP3* ceRNA axis and promote mitophagy. This process contributes to APEC immune evasion and systemic infection. This study provides new insights into the core mechanisms by which OMVs enable APEC to evade innate immune surveillance by macrophages.

## Introduction

Avian pathogenic *Escherichia coli* (**APEC**) is an extraintestinal pathogenic *Escherichia coli* (**ExPEC**) that is primarily transmitted via the respiratory and digestive tracts. It can cause localised and systemic infections in poultry, resulting in significant economic losses to the global poultry industry (Ronco et al., 2017; Hu et al., 2022). Owing to its genetic similarity to human ExPEC, APEC possesses considerable zoonotic potential (Mellata, 2013; Reid et al., 2019). To achieve effective infection, APEC utilises its diverse virulence factors to manipulate the host’s defence mechanisms, thereby facilitating its invasion and colonisation (Khairullah et al., 2024). In host–pathogen interactions, macrophages serve as the first line of defense against APEC infection. They also provide an important intracellular niche for APEC survival within the host (Mellata et al., 2003; Zhuge et al., 2019). Macrophages recognize pathogen-associated molecular patterns (**PAMPs**) through pattern recognition receptors (**PRRs**) and initiate antimicrobial immune responses (Li et al., 2016; Pokharel et al., 2023). However, pathogenic bacteria have evolved various strategies to evade host immune surveillance. For example, APEC utilises the UhpAB system to promote the expression of biofilm and stress protein genes, thereby enhancing stress tolerance and evading immune-mediated killing (Yu et al., 2025) Furthermore, ExPEC utilises the PhoP/PhoQ-HlyF system to inhibit the acidification of phagocytic lysosomes and disrupt their membrane structure, thereby enhancing its survival and replication capacity within macrophages (Zhuge et al., 2018). These complex intracellular escape mechanisms enable APEC to evade host immune killing. Therefore, understanding the molecular mechanisms underlying APEC immune evasion is important for elucidating its persistent infection.

During host colonisation and infection, pathogens secrete outer membrane vesicles (**OMVs**), which are spherical lipid bilayer nanoparticles containing various virulence factors (Avila-Calderón et al., 2021; Magaña et al., 2024). Acting as key vectors for cross-membrane communication, they play a dual role in triggering the host immune response and facilitating pathogen pathogenesis. On the one hand, the large quantities of immunostimulatory cargo carried by OMVs trigger host recognition and responses. For example, OMVs secreted by APEC are recognised by the TLR4 receptor on macrophages, activating signalling pathways that lead to the release of pro-inflammatory cytokines (Wang et al., 2023). Furthermore, the lipopolysaccharides and peptidoglycans abundant in OMVs can activate the host intracellular receptor NOD1, thereby promoting autophagy and the transmission of inflammatory signals (Groeger and Meyle, 2019; Li and Shang, 2024). On the other hand, pathogenic bacteria also actively utilise OMVs to deliver effector molecules to host cells, with the aim of disrupting cellular function and mediating immune evasion. Previous studies have shown that OMVs can carry virulence proteins that target organelles such as mitochondria and the endoplasmic reticulum. For example, OMVs secreted by *Neisseria gonorrhoeae* (***N. gonorrhoeae*)**, urinary tract pathogenic *Escherichia coli* and *Pseudomonas aeruginosa* (***P. aeruginosa***) cause mitochondrial dysfunction, triggering inhibition of host protein synthesis and inducing mitochondrial apoptosis (Bielaszewska et al., 2013; Deo et al., 2018; Pin et al., 2023). Furthermore, APEC OMVs can induce endoplasmic reticulum stress-mediated autophagy stasis in chicken macrophages (**HD11**), thereby promoting their intracellular survival and persistent infection (Cui et al., 2026). OMVs carry a variety of virulence factors and pathogen-associated molecular patterns, allowing them to modulate the host innate immune response. However, how APEC-derived OMVs regulate host immunity and evade immune clearance remains poorly understood. Their role in promoting systemic infection also requires further investigation.

Upon entering host cells, pathogens and their secreted virulence factors can disrupt the structure and function of host organelles. These changes may create a cellular environment that supports nutrient acquisition, immune evasion, and pathogen dissemination within the host (Kellermann et al., 2021). Among these, the complex integration of mitochondria into host cellular processes makes them a key target during pathogen infection (Tiku et al., 2020). Recent studies have reported that Hcp2a, an effector protein of the T6SS in APEC, induces mitochondrial dysfunction in chicken embryo fibroblasts (**DF-1**). This dysfunction leads to the release of large amounts of reactive oxygen species (**ROS**) from mitochondria (Lu et al., 2024). The large amounts of ROS and mitochondrial DNA released as a result of mitochondrial damage can activate the NLRP3 inflammasome and the expression of inflammatory cytokines such as IL-1β and IL-6 (He et al., 2025). There is crosstalk between the inflammasome and mitophagy; to prevent excessive inflammation from damaging the cell, host cells clear damaged mitochondria via the mitophagy pathway to restore homeostasis (Gupta et al., 2026). However, this defence mechanism is often exploited by pathogens. For example, *P. aeruginosa* utilises mitophagy to suppress ROS production and the activation of the NLRC4 inflammasome caused by mitochondrial damage, thereby evading host defence mechanisms (Jabir et al., 2015). Furthermore, *Mycobacterium bovis* (***M. bovis***) and *Staphylococcus aureus* (***S. aureus***) promote their intracellular survival by inducing mitophagy via the PINK1-Parkin pathway, whilst *Salmonella typhimurium* (***S. Typhimurium***) SseJ induces PHB2-mediated mitophagy (Song et al., 2022; Xu et al., 2023; Sun et al., 2025). Mitophagy plays a crucial role in the evasion of host immune killing by pathogenic bacteria. However, it remains unclear whether APEC-derived OMVs induce mitophagy and use this process to promote intracellular survival. Long non-coding RNAs (**lncRNAs**) and microRNAs (**miRNAs**) are non-coding RNA molecules that lack protein-coding capacity (St. Laurent et al., 2015; dos Santos et al., 2022). Increasing evidence indicates that lncRNAs can act as “molecular sponges” by binding to specific miRNAs. This interaction relieves the inhibitory effects of miRNAs on their target mRNAs and forms an “lncRNA–miRNA–mRNA” regulatory axis (Salmena et al., 2011). During the host response to pathogenic bacterial infection, competitive endogenous RNA (**ceRNA**) networks regulate several important biological processes. These processes include innate immune activation, cellular autophagy, and mitochondrial quality control. For example, during APEC infection, the gga-miR-20a-5p/*PTEN* axis plays an important role in regulating host autophagy. It also affects subsequent immune and inflammatory responses (Sun et al., 2024). In a fish model of bacterial infection, the lncRNA 432-miR-21-y-DAPK2 network mediates the loss of mitochondrial membrane potential (**ΔΨm**) and apoptosis. This process contributes to mitochondrial dysfunction (Han et al., 2025). Tumour necrosis factor-α-induced protein 3 (**TNFAIP3**) is a key ubiquitin-editing protein that maintains host immune homeostasis. It suppresses inflammatory responses mainly by negatively regulating signalling pathways such as NF-κB (Shembade et al., 2010). Studies have shown that the central role of *TNFAIP3* in regulating mitophagy and organelle homeostasis is becoming increasingly evident. For example, in a model of nucleus pulposus cell degeneration, it maintains the homeostasis of the mitochondrial network by promoting protective mitophagy (Peng et al., 2022). The role of *TNFAIP3* in inflammation and autophagy regulation has been widely established. However, its role in ceRNA-mediated regulation of mitophagy during pathogen infection remains unclear.

In this study, we combined *in vivo* and *in vitro* infection models to elucidate the molecular mechanism by which OMVs secreted by APEC mediate immune evasion by inducing mitophagy in HD11 cells. Our transcriptomic sequencing data were deposited in the NCBI SRA database (Submission ID: SUB16323207). Differential expression analysis and bioinformatic prediction identified a novel lncRNA, MSTRG.11745.1. This lncRNA was markedly induced by APEC OMVs stimulation and may be involved in the regulation of mitophagy. MSTRG.11745.1 functions as a ceRNA and acts as a molecular sponge for gga-miR-15b-5p. This reduces the binding of gga-miR-15b-5p to its target *TNFAIP3* and relieves the inhibitory effect on *TNFAIP3* expression. The upregulation of *TNFAIP3* further triggers mitophagy and reduces intracellular ROS levels, thereby significantly promoting the survival and proliferation of APEC within the host. This study identifies the “lncRNA–miRNA–mRNA” regulatory axis involved in APEC OMVs-induced mitophagy for the first time. It provides new insights into the pathogenic mechanisms of APEC. It also provides a theoretical basis for developing novel strategies for APEC prevention and control.

## Materials and methods

### Ethics statement

All animal experimentation protocols were conducted in accordance with the Anhui Provincial Regulations on the Management of Laboratory Animals and were approved by the Animal Experimentation Ethics Committee of Anhui Agricultural University (KJLLXM2025104). This study established clear humane endpoints for laboratory animals: should an animal exhibit significant weight loss (≥ 10%), persistent reduction in food intake, reduced mobility, lethargy or other irreversible signs of distress, the experiment would be terminated immediately and the animal euthanised in accordance with ethical guidelines to minimise suffering.

### *Reagents*

Carbonyl cyanide 3-chlorophenylhydrazone (CCCP, HY-100941), Mitochondrial division inhibitor1 (Mdivi-1, HY-15886), and Mito-TEMPO (HY-112879) were purchased from MedChemExpress (New Jersey, USA). Rabbit anti-LC3 polyclonal antibody (14600-1-AP), mouse anti-β-actin monoclonal antibody (66009-1-Ig), and mouse anti-HSP60 monoclonal antibody (66041-1-Ig) were purchased from Proteintech (Wuhan, China). Rabbit anti-TOM20 polyclonal antibody (T55527S) and rabbit anti-TIMM23 polyclonal antibody (PH3553S) were purchased from Abmart (Shanghai, China). Mouse anti-LAMP1 monoclonal antibody (222466), HRP-conjugated goat anti-rabbit IgG (511203), and HRP-conjugated goat anti-mouse IgG (511103) were purchased from Zen-BioScience (Sichuan, China). Cy3-conjugated goat anti-mouse IgG (A0521), FITC-conjugated goat anti-rabbit IgG (A0562), and FITC-conjugated goat anti-mouse IgG (A0568) were purchased from Beyotime (Shanghai, China).

### *Bacterial strains and culture conditions*

The clinical strain APEC81 (serotype O166:H45) used in this study was isolated from the lung of a chicken that had died from symptoms of *Escherichia coli* septicaemia in Anhui, China (Fu et al., 2021). The OMVs secretion-deficient strain APEC81Δ*ypjA* (Li et al., 2024) is housed at the Anhui Provincial Key Laboratory of Veterinary Pathology and Disease Control (Anhui, China), whilst APEC81Δ*ldcA* was constructed using homologous recombination. The full length sequence of MSTRG.11745.1 was shown in the Table S1.

APEC81 (**WT**), APEC81 (**Δ*ypjA*, Δ*ldcA)*** were inoculated into antibiotic-free Luria-Bertani (**LB**) broth or LB agar and cultured at 37°C. In LB broth, the culture was subcultured at a ratio of 1:100 into fresh LB broth and incubated under shaking conditions at 180 rpm. Bacterial density was estimated by measuring the optical density at 600 nm (**OD_600_**), where an OD_600_ of 1.0 corresponded approximately to 1 × 10⁹ CFU/mL, whilst agar plates were used for colony isolation and viable cell counting.

### *Construction and validation of bacterial strains, mutants in the functional region of the ldcA gene*

The gene sequence of *ldcA* functional region was provided by NCBI (Gene ID: 945756). Mutant strains were constructed by knockdown using a lambda red recombinase system(Datsenko and Wanner, 2000), the chloramphenicol resistance fragment amplified using pKD3 plasmid as the template, gel recovered and transformed into APEC81 cells containing pKD46 plasmid using a Gene Pulser Xcell (Bio Rad, Hercules, CA, USA) at 200 W and 2.5 kV. The pCP20 plasmid was utilized to cure pKD3 and the mutant strain was named APEC81Δ*ldcA*, after being identified by real-time quantitative PCR (**RT-qPCR**). Primers are shown in detail in Table S2.

### *Plasmid construction*

The low-copy plasmids pmirGLO-*TNFAIP3*-3′UTR-WT/MUT and pmirGLO-MSTRG11745.1-WT/MUT were purchased from Qingke Biotechnology; the pcDNA3.1-MSTRG11745.1 and pcDNA3.1 empty vector plasmids were purchased from General Biotechnology.

### *Cell culture and transfection*

The HD11 cells and the HEK293T human embryonic kidney cells (**293T**) used in this study were both maintained by the Key Laboratory of Veterinary Pathobiology and Disease Control (Anhui, China). Cells were cultured in DMEM (VivaCell, Shanghai, China) supplemented with 10% foetal bovine serum (ExCell Bio, Shanghai, China) and 1% antibiotic solution (containing 100 U/mL penicillin and 100 μg/mL streptomycin). Cells were incubated at 37°C in a humidified environment containing 5% CO₂. Lipofectamine 3000 (Thermo Fisher, MA, USA) and Opti-MEM medium (Gibco, California, USA) were used to transfect to 293T cells or HD11 cells with plasmid DNA (pmirGLO-*TNFAIP3*-3′UTR-WT/MUT, pmirGLO-MSTRG11745.1-WT/MUT, pcDNA3.1-MSTRG11745.1, pcDNA3.1 empty vector). In experiments involving chemical inhibitors, cells were pre-treated with Mdivi-1 (20 μM, 2 h), Mito-TEMPO (10 μM, 2 h) or CCCP (10 μM, 2 h) prior to subsequent experiments.

### *Extraction and purification of OMVs*

In this study, OMVs were extracted from the culture supernatant of APEC81 using a method adapted from Welsh J A et al. (2024). Firstly, the APEC81 strain was cultured in LB broth medium until OD_600_ = 1. The supernatant was collected and filtered successively with 0.45-μm and 0.22-μm filters. Subsequently, the filtrate was concentrated using 100-kDa ultrafiltration tubes (Millipore, MA, USA), and then centrifuged at 4°C with a relative centrifugal force of 39,000 × *g* for 3 h for crude extraction. The OMVs precipitate was resuspended in filtered 10 mM 4-(2-hydroxyethyl)-1-piperazineethanesulfonic acid (HEPES) buffer (pH 7.4) containing 45% (w/v) OptiPrep (Sigma-Aldrich, MO, USA), 0.85% NaCl. To further purify the OMVs, the crude OMVs extract was added onto a 40% (bottom) and 25% (top) gradient OptiPrep solution and centrifuged at 298,700 × *g* for 6 h at 4°C. After centrifugation, the fraction corresponding to the 28–31% OptiPrep layer was collected, diluted eightfold with sterile HEPES buffer, and centrifuged again at 39,000 × *g* for 3 h at 4°C to remove residual OptiPrep. Finally, the precipitate on the centrifuge tube was suspended in sterile phosphate-buffered saline (**PBS**) to obtain the purified OMVs, which was stored at −80°C. The purified OMVs were quantified using a BCA protein assay kit (Sparkjade, Shandong, China) and stored at −80°C for future use.

### *Nanoparticle tracking analysis*

Nanoparticle tracking analysis was performed to determine the concentration and size distribution of OMVs using a ZetaView PMX 110 nanoparticle tracking analyzer (Particle Metrix, Germany). Measurements were performed using a 405-nm laser to assess particle size distribution and concentration. Before analysis, the OMVs samples were diluted with PBS to a final concentration of 1 × 10⁷ to 1 × 10⁹ particles/mL. The diluted samples were then analyzed to determine the size distribution and concentration of OMVs.

### *OMVs Endocytosis and gentamicin-protection assay*

HD11 cells were seeded at a density of 2 × 10⁵ cells per well in a 12-well culture plate and cultured for 16 h. The culture medium was then removed, and the cells were treated with OMVs (100 μg/mL) diluted in DMEM for the indicated times. In the fluorescence assay to observe OMVs internalisation, OMVs were labelled with DiO dye (10 μM) (Yeasen, Shanghai, China) according to the manufacturer’s instructions. For the gentamicin protection assay, the WT and Δ*ypjA* were cultured in fresh LB medium to an OD_600_ of 1.0, and used to infect HD11 cells at an MOI of 100 for 2 h, with three replicate wells set up. Upon completion of infection, the culture medium was discarded, and the cells were washed three times with PBS; this time point was designated as 0 h post-infection. Fresh DMEM medium containing 100 μg/mL gentamicin was then added to continue the culture, thereby killing the extracellular bacteria. Culture was continued for 1 h, 2 h and 3 h, after which the cells were lysed with 0.5% Triton X-100 (Solarbio, Beijing, China) for 10 min. The cell lysates were serially diluted and spread onto LB agar plates; after incubation overnight at 37°C, the CFU were counted. All experiments were repeated independently three times to ensure the reliability of the results.

### *Transmission electron microscopy (TEM) observation*

The isolated and purified OMVs were analysed by TEM. The OMVs, stored at –80°C, were thawed and thoroughly mixed, then dispensed onto a copper grid. They were stained with 2% phosphotungstic acid and, after drying, the morphology of the OMVs was observed using a transmission electron microscope (HITACHI, Tokyo, Japan).

The cell pellets from HD11 cells treated with WT and OMVs were collected, fixed overnight at 4°C in a 2.5% glutaraldehyde solution (Solarbio, Beijing, China), and then fixed for 1–2 h in a 1% osmium tetroxide solution. Subsequently, the samples were dehydrated using an ethanol gradient and transferred to pure acetone; after routine resin embedding, they were polymerised at 70°C. Sections of 70–90 nm thickness were cut using an ultramicrotome (LEICA, Wetzlar, Germany); following double staining with uranyl acetate and lead citrate, the mitochondrial structures were observed using a transmission electron microscope.

### *ROS And δψm assays*

HD11 cells (1 × 10⁵ cells/well) were seeded in 24-well culture plates. Following treatment of the cells with WT, Δ*ypjA* or OMVs, the subsequent procedures were carried out. For the intracellular ROS assay, the ROS fluorescent probe DHE (Solarbio, Beijing, China), diluted 1:800 with fresh DMEM, was added to the cells. The cells were incubated for 30 min in a 37°C incubator, washed three times with PBS, and imaged using an inverted fluorescence microscope (Olympus, Tokyo, Japan). For the *Δψm* assay, following the instructions for the Mitochondrial Membrane Potential Assay Kit with JC-1 (Solarbio, Beijing, China), The prepared JC-1 working solution was added to the treated cells and incubate at 37°C in a cell culture incubator for 20 min. The supernatant was removed and wash twice with JC-1 staining buffer. Two millilitres of cell culture medium were added, and images were captured using an inverted fluorescence microscope (Olympus, Tokyo, Japan). Quantitative analysis of the fluorescence images was performed using ImageJ, with the proportion of mitochondrial membrane depolarisation assessed by analysing the relative ratio of red to green fluorescence.

### *Immunofluorescence staining and fluorescence microscopy analysis*

In the indirect immunofluorescence assay, 1 × 10⁵ cells were seeded onto a 24-well culture plate fitted with round glass coverslips (NEST, Jiangsu, China). 6 h after treatment with OMVs, if mitochondria were to be labelled, live cells were stained using Mito-Tracker Red CMX-ROS (Beyotime, Shanghai, China). Following staining, the cells were fixed with 4% paraformaldehyde at room temperature for 15 min and washed three times with PBS. Subsequently, cells were permeabilized with 0.1% Triton X-100 (Solarbio, Beijing, China) for 10 min, then wash the cells three times with PBS. cells were blocked containing 5% bovine serum albumin (**BSA**) at 37°C for 1 h, followed by overnight incubation with the appropriate primary antibody at 4°C. The cells were washed three times with PBS for 5 min each time; they were then incubated with the corresponding fluorescent secondary antibody (diluted 1:500 in PBS) at room temperature for 1 h. After three washes with PBS, the slides were sealed with an anti-fluorescence quenching mountant (containing DAPI) (Sparkjade, Shandong, China). Image acquisition was performed using a laser confocal microscope (LEICA, Wetzlar, Germany). Line-scan analysis of the confocal images was carried out using ImageJ to extract fluorescence intensity distribution curves for each fluorescence channel, in order to assess the spatial distribution and colocalisation of different fluorescence signals. Colocalisation line graph were plotted using GraphPad Prism 8 software.

### *Western blot analysis*

Total cellular proteins were extracted using RIPA lysis buffer containing a mixture of protease inhibitors (Epizyme Biotech, Shanghai, China). After lysis on ice for 15 min, the lysate was collected into 1.5 mL microcentrifuge tubes and centrifuged at 12,000 × *g* for 10 min at 4°C. The supernatant was mixed with 5 × SDS loading buffer (Epizyme Biotech, Shanghai, China) and boiled at 100°C for 15 min to denature the proteins. Equal volumes of protein samples were separated by SDS-PAGE and transferred to a PVDF membrane (Millipore, MA, USA). The membrane was blocked with 5% skimmed milk at room temperature for 2 h, then incubated overnight at 4°C with the specified primary antibody. After washing three times with TBST buffer for 7 min each time, the membrane was incubated with the HRP-conjugated secondary antibody at room temperature for 1 h. After washing with TBST three more times, the membrane was developed using an ultra-sensitive enhanced chemiluminescence (**ECL**) kit (Epizyme Biotech, Shanghai, China), and the target bands were visualised using a chemiluminescence imaging system. The grey-scale values of the bands were quantitatively analysed using ImageJ software. β-actin was used as an internal reference protein to calculate the relative expression levels of the target proteins.

### *Quantitative real-time PCR (qPCR)*

To analyse mitochondrial DNA (**mtDNA**) levels, this study utilised HD11 treated with WT, Δ*ypjA* and OMVs. Cellular DNA was extracted using a cellular genomic DNA extraction kit (Sparkjade, Shandong, China) in accordance with the manufacturer’s instructions. To the reaction mixture, 5 μL of 2 × SYBR Green qPCR Mix (Sparkjade, Shandong, China), 1 μL of DNA, and 0.5 μL each of the COXII (mitochondrial DNA) and RPL13A (genomic DNA) primers were added to perform quantitative real-time PCR in a total volume of 10 µL. Three replicates were performed for each sample, and the relative differences in mitochondrial DNA and genomic DNA were calculated using the 2^-ΔΔCt^ method.

To detect the relative expression levels of lncRNA MSTRG.11745.1 and mRNA *TNFAIP3*, total cellular RNA was extracted using the SPARKeasy Tissue/Cell RNA Rapid Extraction Kit (Sparkjade, Shandong, China). Subsequently, reverse transcription of the extracted RNA was performed according to the instructions for the EasyScript® One-Step gDNA Removal and cDNA Synthesis SuperMix Kit (TransGen Biotech, Beijing, China). GAPDH was used as the internal control for the detection of MSTRG.11745.1 expression, whilst β-actin was used as the internal control for the detection of *TNFAIP3* expression. A 10 µL total reaction mixture was prepared for RT-qPCR. Three replicates were performed for each sample, and relative gene expression was quantified using the 2^-ΔΔCt^ method. The relevant primers are shown in Table S2.

### *Animal infection model*

To investigate the role of OMVs-induced mitophagy in the course of APEC infection *in vivo*, this study established a systemic infection model in chicks and systematically assessed pathological damage and bacterial colonisation levels in organs such as the trachea, lungs, liver and spleen (Antão et al., 2009; Li et al., 2023). The one-day-old Romann laying hens used in this study were purchased from Anhui Anqin. Firstly, 7-day-old chicks were randomly assigned to the following groups: the WT group, the Δ*ypjA* group, the WT+Mdivi-1 group, the Mdivi-1 group and the sterile PBS control group, with 10 chicks in each group. The sample size was determined based on previous research experience and preliminary experimental results to ensure sufficient statistical power to detect the expected effects (power≥0.8, significance level α = 0.05). Chicks were inoculated intratracheally with a bacterial dose of 1 × 10⁹ CFU per bird using sterile syringes, and the challenged chicks were reared until day 6. For the WT + Mdivi-1 and Mdivi-1 groups, chicks received intraperitoneal injections of Mdivi-1 (25 mg/kg) starting 24 h after infection and continuing daily until day 6. The Mdivi-1 dose was selected based on previous reports of its *in vivo* use as a pharmacological inhibitor of mitochondrial fission and mitophagy, together with the results of preliminary experiments (Gao et al., 2024). The WT and Δ*ypjA* groups received an equivalent volume of PBS. On day 6 post-infection, the chicks were euthanised. Tissues were harvested for observation of pathological changes. A 0.1 g sample of each tissue was weighed, ground using a tissue grinder, centrifuged at low speed, and the resulting PBS supernatant was serially diluted and spread onto MacConkey agar plates to record differences in bacterial load. The trachea, lung (left lobe), liver and spleen were fixed in 4% paraformaldehyde and prepared as paraffin-embedded sections. Following haematoxylin and eosin (**H&E**) staining, histopathological changes were observed under a light microscope.

### *Transcriptome sequencing of HD11 cells*

HD11 cells from the control (DMEM) and APEC OMVs-treated groups were collected by scraping after 6 h of treatment. The cells were immediately snap-frozen in liquid nitrogen and stored at −80°C. The samples were then transported on dry ice to Qingke Biotechnology (Beijing, China) for transcriptome sequencing using an Illumina® sequencing platform.

### *Bioinformatics analysis*

Gene expression levels were calculated using the fragments per kilobase of exon per million mapped fragments (FPKM) method. These data were used for differential expression analysis. DElncRNAs were identified using the following criteria: |log_2_FoldChange| ≥ 1 and *P* ≤ 0.05. Potential cis- and trans-target genes of the lncRNAs were then predicted. The prediction was based on their genomic locations and expression correlations with protein-coding genes. GO and KEGG enrichment analyses were performed to identify potential target genes related to mitochondrial function, mitochondrial quality control, and mitophagy. Co-expression analysis was further performed based on the lncRNA and mRNA expression levels in the RNA-seq samples. mRNAs showing a significant positive correlation with DElncRNAs were selected using the criteria of Pearson *r* ≥ 0.90 and *P* ≤ 0.05. Candidate lncRNAs potentially involved in APEC OMVs-induced mitophagy were then identified based on mitophagy-related target genes and their functional enrichment results. Finally, several candidate lncRNAs were experimentally validated to further evaluate their potential roles in APEC OMVs-induced mitophagy.

### *Construction of the ceRNA network*

The online tools TargetScan, RNAHybrid and miRanda v3.3a were utilised to predict the targeting relationships between differentially expressed lncRNAs and miRNAs, and between miRNAs and mRNAs, under APEC OMVs treatment conditions. An lncRNA-miRNA-mRNA interaction network was constructed using the R programming language. Only differentially expressed RNAs that satisfied the prediction criteria and exhibited inverse expression patterns were included in the ceRNA network.

### *MiRNA Mimics and siRNA transfection*

The gga-miR-15b-5p mimics, si-*TNFAIP3* and si-MSTRG.11745.1 were all designed and purchased from Qingke Biotechnology; The sequences are listed in Table S3. Transfection was performed using Lipofectamine 3000 (Thermo Fisher, MA, USA), following the manufacturer’s instructions.

### *Dual-luciferase assay*

The online tools TargetScan, RNAHybrid and miRanda v3.3a were utilised to predict the miRNAs that bind to MSTRG.11745.1, as well as their target mRNAs. A dual-luciferase reporter assay was conducted to validate the binding relationships between gga-miR-15b-5p and MSTRG.11745.1, and between gga-miR-15b-5p and *TNFAIP3*, respectively. First, gga-miR-15b-5p mimics were co-transfected with pmirGLO-*TNFAIP3*-3′UTR-WT/MUT and pmirGLO-MSTRG11745.1-WT/MUT, respectively, into 293T cells. After 24 h of culture, the cell culture medium was aspirated, and 300 μL of Dual Luciferase Reporter Gene Assay Cell Lysis Buffer (Beyotime, Shanghai, China) was added to the 12-well plates to thoroughly lyse the cells. Eighty microlitres of cell lysate were transferred to an white opaque 96-well plate, and, in accordance with the instructions for the Dual Luciferase Reporter Gene Assay Kit (Yeasen, Shanghai, China), the firefly and Renilla luciferase luminescence signals were detected sequentially on a multi-functional microplate reader equipped with a chemiluminescence module. The relative luciferase activity of fireflies was calculated using the formula given in the instructions.

### *Statistical analysis*

All values are expressed as the mean ± SEM of several individual samples. All data are derived from at least three independent experiments. Statistical analysis was performed using Student’s t-test and two-way analysis of variance (ANOVA), with two-tailed tests. All statistical analyses were performed using GraphPad Prism 8 software. Statistical significance is indicated according to the following criteria: *P < 0.05, ** *P* < 0.01, *** *P* < 0.001.

## Results

### *APEC Enhances infection and induces autophagy through the secretion of OMVs*

**Fig. 1:**
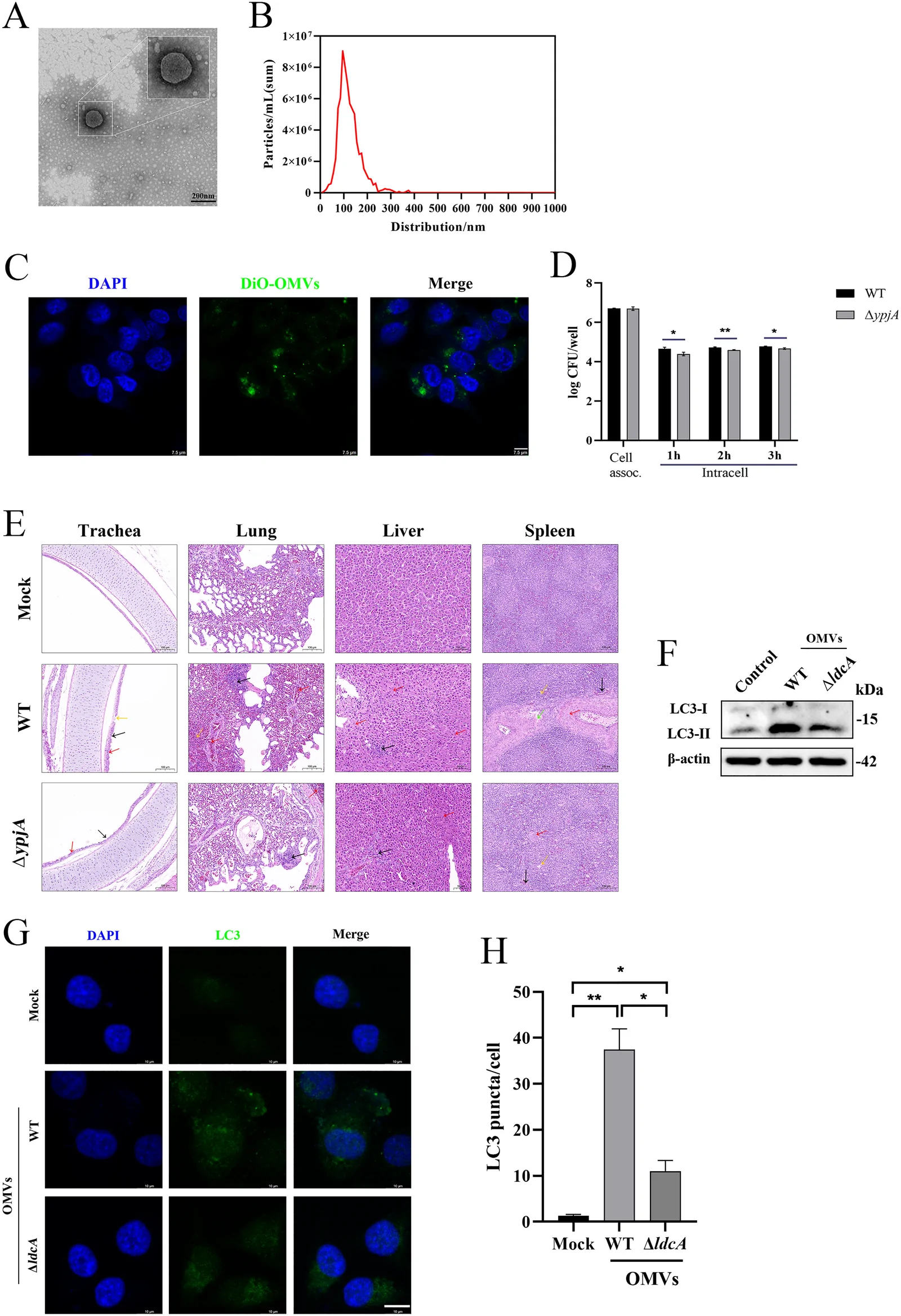
**APEC enhances infection and induces autophagy through the secretion of OMVs.** (A, B) Analysis of the morphology and concentration of APEC OMVs. Scale bar = 200 nm. (C) Laser confocal microscopy showing DiO-labelled OMVs entering HD11 cells; no structural signals were observed in the dye control group. Data represent one of three independent experiments. Scale bar = 7.5 μm. (D) Intracellular survival of WT and Δ*ypjA* following infection of HD11 cells at an MOI of 100. The intracellular bacterial load in Δ*ypjA* was significantly lower than that in the wild-type strain. (n = 3). (E) H&E staining of chick trachea, lung, spleen and liver tissues 6 days post-infection with WT and Δ*ypjA*. Trachea: deformation and desquamation of epithelial cells (black arrow), inflammatory cell infiltration in the lamina propria (red arrow), and marked shedding and breakage of cilia (yellow arrow); Lung: inflammatory cell infiltration in the pulmonary stroma (black arrow), marked thickening of the pulmonary interstitium, and a significant reduction in airspace area (yellow arrow), with marked dilation and congestion of capillaries (red arrow); Liver: inflammatory cell infiltration (black arrow), blurred hepatocyte margins and markedly abnormal cell morphology (red arrow); Spleen: dilated red pulp accompanied by haemorrhage and diffuse inflammatory cell infiltration (black arrow), reduced lymphocyte numbers (yellow arrow), and markedly thickened blood vessel walls (red arrow) accompanied by vacuolisation (green arrow). Scale bar = 100 µm or 50 µm. (F) LC3-II conversion in HD11 cells treated with Δl*dcA* OMVs. Compared with WT OMVs, the extent of LC3-II conversion in HD11 cells treated with Δ*ldcA* OMVs was reduced. (n = 3). (G) LC3 dot aggregation in HD11 cells treated with Δ*ldcA* OMVs. Compared with WT OMVs, LC3 dot aggregation was reduced in HD11 cells treated with Δ*ldcA* OMVs. Data represent one of three independent experiments. Scale bar = 10 µm. (H) Quantitative analysis was performed using ImageJ. n represents 3 biological replicates, and individual data points represent independent culture systems. Data are presented as means ± SD. Statistical significance was assessed using Student’s *t*-test or two-way ANOVA with Sidak’s multiple-comparisons correction. (* *P* < 0.05, ** *P* < 0.01, *** *P* < 0.001).

To investigate the effects of OMVs on APEC infection and intracellular survival, we first isolated and purified OMVs secreted by APEC. Transmission electron microscopy showed that the OMVs exhibited a typical spherical structure with a bilayer membrane (Fig. 1A). The particle size mainly ranged from 20 to 400 nm (Fig. 1B), which was consistent with the typical characteristics of OMVs. The presence of the OMV markers OmpA and OmpF was confirmed by Western blotting in our previous study (Cui et al., 2026). Furthermore, OMVs were labeled with the fluorescent dye DiO to assess their internalization by HD11 cells. Confocal microscopy revealed distinct green fluorescence signals in the cytoplasm of HD11 cells, indicating efficient internalization of OMVs by the cells (Fig. 1C). Next, HD11 cells were infected with WT or Δ*ypjA* at an MOI of 100, and intracellular bacterial survival was determined at 1, 2, and 3 h after infection. Compared with WT, Δ*ypjA* showed significantly lower intracellular survival at 1 h (*P* < 0.05), 2 h (*P* < 0.01), and 3 h (*P* < 0.05) (Fig. 1D). *In vivo*, a chick infection model was established to assess tissue pathology. Both WT- and Δ*ypjA*-infected chicks exhibited obvious histopathological damage compared with the control group (Fig. 1E). In the trachea, both groups exhibited epithelial cell deformation and desquamation, as well as inflammatory cell infiltration in the lamina propria; in the WT group, cilia were markedly shed and broken. In the lungs, marked inflammatory cell infiltration was observed in the stroma; in the WT group, the interstitial spaces were markedly thickened, the airspace area was significantly reduced, and the capillaries were markedly dilated and congested. In the liver, infected chickens exhibited hepatocyte degeneration and inflammatory cell infiltration, which were more pronounced in the WT group. In the spleen, both groups of infected chickens showed an indistinct boundary between the white pulp and red pulp, a reduction in lymphocyte numbers, and expansion of the red pulp accompanied by haemorrhage and diffuse inflammatory cell infiltration; in the WT group, this was further accompanied by significant thickening of the vascular wall (media) with vacuolisation.

To investigate the effect of APEC-secreted OMVs on the induction of autophagy in HD11 cells, HD11 cells were treated with APEC OMVs and their autophagy levels were assessed. The results showed that, compared with the control group (treated with DMED), there was marked LC3 dot aggregation following treatment with APEC OMVs. To investigate, as a preliminary step, whether the autophagy response induced by APEC OMVs depends on the NOD1 signalling pathway, we used OMVs derived from the Δ*ldcA* mutant for validation. The peptidoglycan receptor NOD1 specifically recognises peptidoglycan fragments terminating in meso‑diaminheptanedioic acid (meso‑DAP), thereby activating downstream autophagy signalling pathways (Lenz et al., 2017); however, Δ*ldcA* is unable to produce peptidoglycan containing a meso‑DAP terminal residue, thereby evading NOD1-dependent autophagy. Western blot results revealed that OMVs derived from Δ*ldcA* still activated autophagy in HD11 cells, albeit at lower levels than those secreted by the WT strain (Fig. 1F). Furthermore, WT OMVs significantly increased LC3 puncta formation compared with the Mock group (*P* < 0.01). This increase was significantly reduced by Δ*ldcA* OMVs compared with WT OMVs (*P* < 0.05), although LC3 puncta formation remained higher than that in the Mock group (*P* < 0.05) (Fig. 1G, H). Therefore, in addition to activating the NOD1-dependent autophagy pathway, APEC OMVs also activate autophagy via an alternative mechanism.

### *APEC Induces mitochondrial fragmentation in HD11 cells via the secretion of OMVs*

**Fig. 2:**
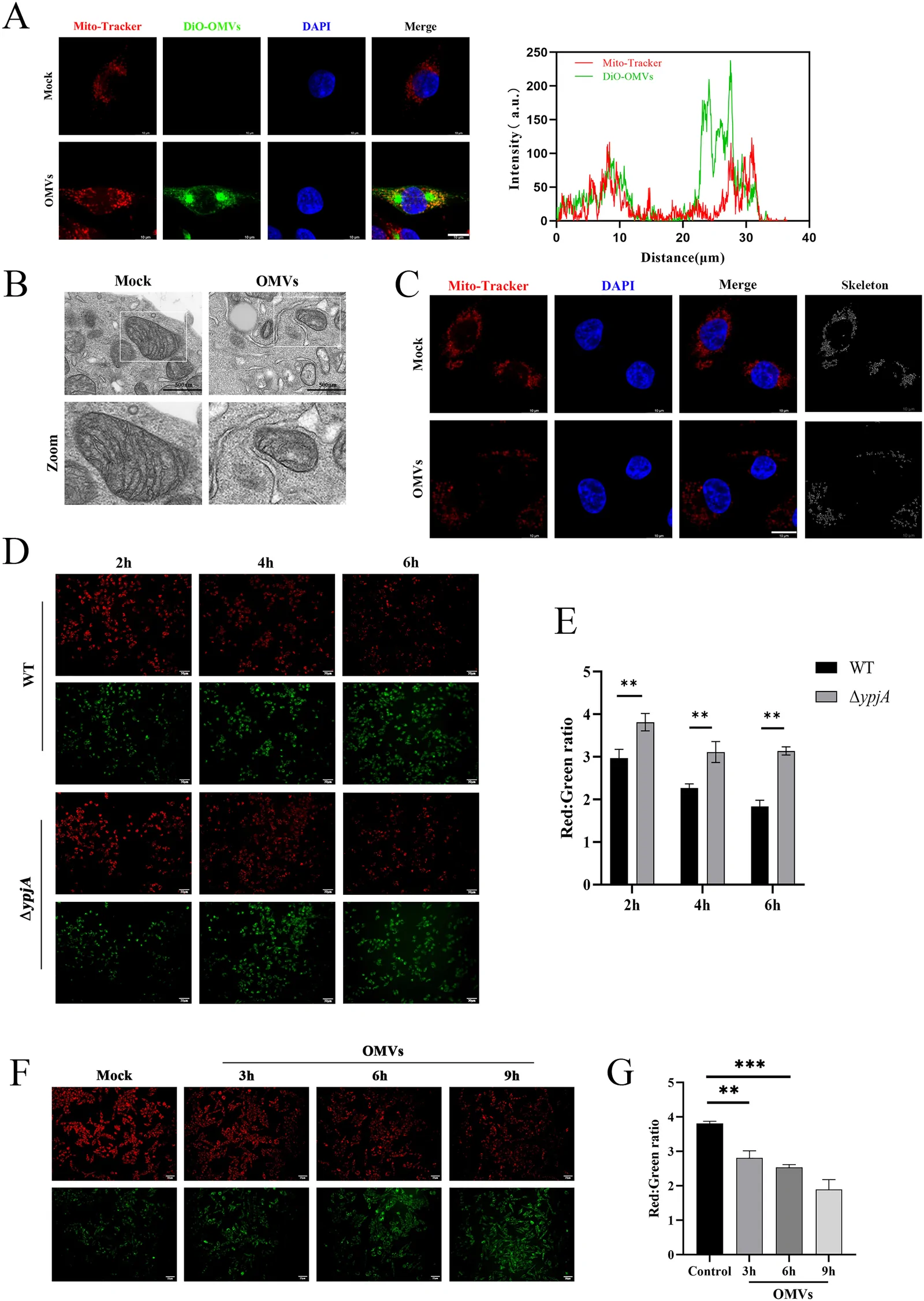
**APEC induces mitochondrial damage in HD11 cells via the secretion of OMVs.** (A) APEC OMVs colocalise with mitochondria. DiO—OMVs were co-cultured with HD11 cells for 6 hours; mitochondria were labelled with Mito-Tracker Red CMXRos and imaged using a laser confocal microscope. Data represent one of three independent experiments. Scale bar = 10 μm. Co-localisation scatter plots were generated using ImageJ. (B) TEM of HD11 cells treated with OMVs reveals marked swelling of the mitochondrial cristae and an enlargement of the intercristae spaces; in some cases, the cristae have disintegrated, fractured or disappeared; the mitochondrial membranes have ruptured and dissolved, fusing with the matrix. The data are representative of three independent experiments. Scale bar = 500 nm. (C) APEC OMVs reverse mitochondrial network disruption in HD11 cells. Laser confocal microscopy imaging. Data represent one of three independent experiments. Scale bar = 10 μm. Skeletonised images of the mitochondrial network were generated using ImageJ to visualise continuous branches and discrete punctate mitochondria. (D) Mitochondrial membrane potential depolarisation levels were measured in HD11 cells infected with WT and Δ*ypjA* at an MOI of 100 for 2–6 h. Compared with WT, Δ*ypjA* induces a lower degree of mitochondrial membrane potential depolarization. Data represent one of three independent experiments. Scale bar = 50 μm. (F) Results of JC-1 staining of Δψm in HD11 cells treated with APEC OMVs for 0∼9 h. The depolarisation of Δψm increased in a time-dependent manner. Data represent one of three independent experiments. Scale bar = 25 μm. (E, G) Quantitative analysis of fluorescence images was performed using ImageJ software. Data points represent independent culture systems; bar graphs show mean ± standard error, analysed using Student’s t-test or two-way ANOVA with Sidak correction. (** *P* < 0.01, *** *P* < 0.001).

Mitochondria are the organelles within the cell that are most vulnerable to attack by exogenous virulence factors and are closely associated with cellular stress and survival. To investigate the role of APEC in damaging mitochondrial structure and function through the secretion of OMVs, we observed, via laser confocal microscopy, a clear colocalisation between DiO—OMVs and Mito-Tracker (Fig. 2A), indicating that APEC OMVs are associated with mitochondria in HD11 cells to exert their effects; TEM revealed that OMVs-treated cells exhibited evident mitochondrial damage, including cristae disruption, swelling, and membrane rupture compared with control cells (Fig. 2B) (Fig. S1). To further demonstrate the effect of APEC OMVs on the morphology of the mitochondrial network, we examined the mitochondrial network and morphology using laser confocal microscopy following Mito-Tracker staining. Mitochondria in the control group exhibited a highly interconnected tubular network structure, extending throughout the cytoplasm to form a fine reticular or tubular network; following treatment with APEC OMVs, the mitochondria became fragmented, the continuous tubular network structure disappeared, and a large number of discrete, isolated punctate or short rod-like structures appeared (Fig. 2C). Next, Δψm was assessed using JC-1 staining. Following infection with WT or Δ*ypjA*, the membrane potential showed a downward trend over time (2 h, 4 h, 6 h), with the decrease being more pronounced in WT than in Δ*ypjA* (*P* < 0.01) (Fig. 2D, E). Similarly, following treatment with APEC OMVs, the mitochondrial membrane potential showed a time-dependent decline at 3, 6, and 9 h, with significant decreases observed at 3 h (*P* < 0.01) and 6 h (*P* < 0.001). (Fig. 2F, G).

### *APEC Induces mitophagy in HD11 cells via the secretion of OMVs*

**Fig. 3:**
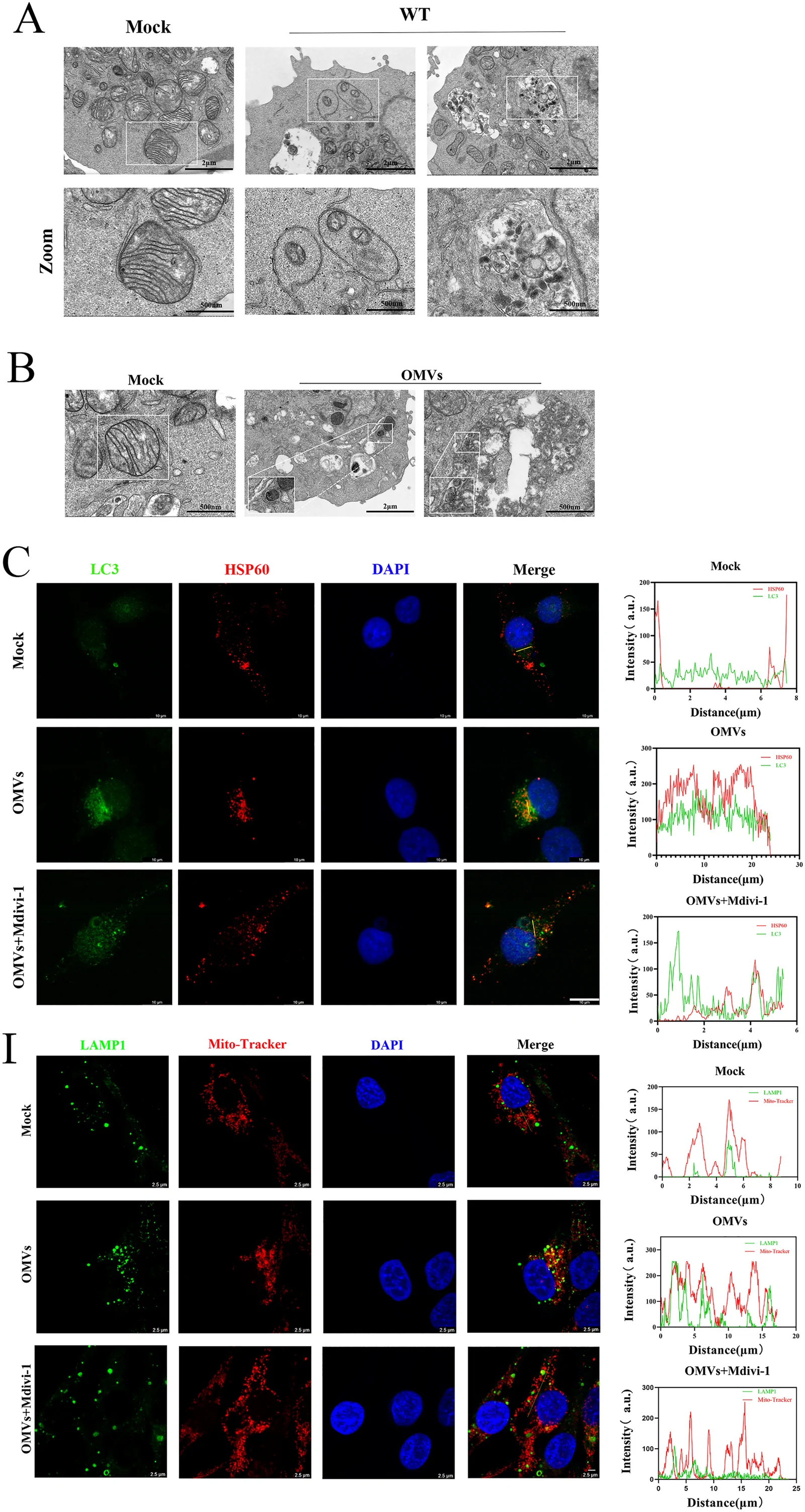
**Mitochondria in HD11 cells are enveloped by autophagosomes and engulfed by lysosomes.** (A, B) Transmission electron micrographs of HD11 cells treated with WT or APEC OMVs. Treatment with WT or APEC OMVs increased the formation of mitochondrial autophagosomes, in which mitochondria were enclosed within autophagosomes and localised to lysosomes. The data are representative of three independent experiments. Scale bar = 2 μm or 500 nm. (C) Analysis of the colocalisation of the mitochondrial markers HSP60 and LC3 in HD11 cells following treatment with APEC OMVs and Mdivi-1. Treatment with APEC OMVs promotes the colocalisation of HSP60 and LC3 in HD11 cells, whilst treatment with Mdivi-1 reduces the extent of their colocalization. Data represent one of three independent experiments. Scale bar = 10 μm. Co-localisation scatter plots were generated using ImageJ. (D) Analysis of the colocalisation of mitochondria and the lysosomal marker protein LAMP1 in HD11 cells following treatment with APEC OMVs and Mdivi-1. Treatment with APEC OMVs promotes the colocalisation of mitochondria and LAMP1 in HD11 cells, whilst treatment with Mdivi-1 reduces the extent of this colocalisation. The data represent one of three independent experiments. Scale bar = 2.5 μm. Co-localisation scatter plots were generated using ImageJ.

**Fig. 4:**
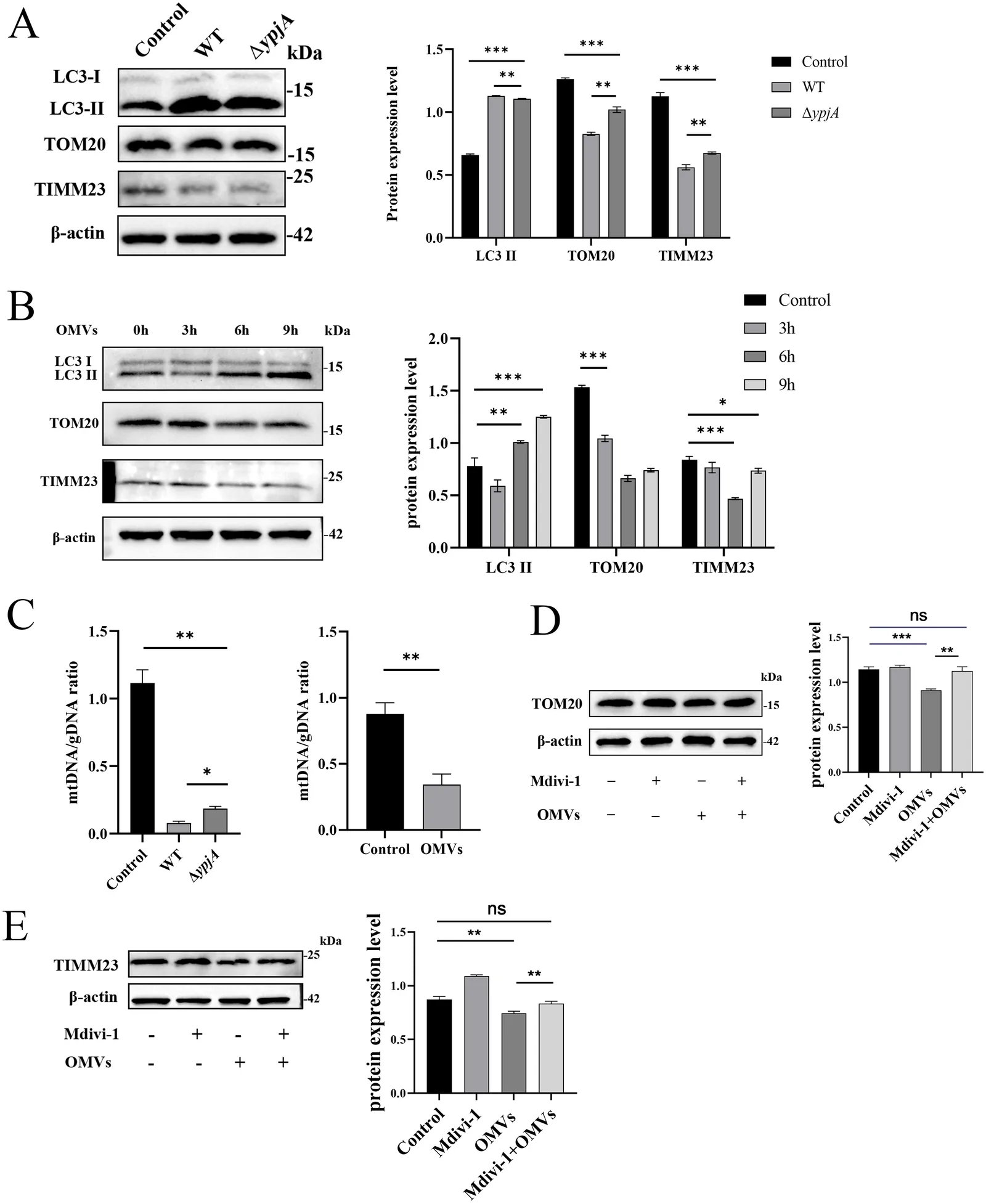
**APEC OMVs induce mitophagy and degradation in HD11 cells.** (A) HD11 cells were infected with WT or Δ*ypjA* strains at an MOI of 100 for 4 h. Changes in LC3-II conversion and the expression of the mitochondrial membrane proteins TIMM23 and TOM20 were examined. Compared with WT infection, Δ*ypjA* infection significantly reduced LC3-II conversion and attenuated the degradation of TIMM23 and TOM20. Protein levels were evaluated by Western blotting and densitometric analysis. (n = 3). (B) Changes in LC3-II conversion and the levels of the mitochondrial membrane proteins TIMM23 and TOM20 in HD11 cells treated with APEC OMVs for 0–9 h. APEC OMVs treatment increased LC3-II conversion and promoted the degradation of TIMM23 and TOM20. Protein levels were evaluated by Western blotting and densitometric analysis. (n = 3). (C) WT, Δ*ypjA*, and APEC OMVs treatments reduced the relative mtDNA levels in HD11 cells. Compared with Δ*ypjA*, WT treatment resulted in a greater reduction in relative mtDNA levels. (n = 3). (D, E) APEC OMVs treatment promoted mitochondrial protein degradation in HD11 cells, whereas Mdivi-1 treatment attenuated this effect. Protein levels were evaluated by Western blotting and densitometric analysis. (n = 3). n represents three biological replicates; data points correspond to independent culture systems; bar graphs show mean values ± standard error; analysis was performed using Student’s t-test (* *P* < 0.05, ** *P* < 0.01, *** *P* < 0.001).

In response to structural and functional damage to mitochondria, cells typically initiate mitophagy to perform quality control and maintain cellular homeostasis. The decrease in Δψm identified in the previous findings serves as a marker of mitochondrial dysfunction and acts as the key trigger for mitophagy. To further investigate whether APEC OMVs induce mitophagy in HD11 cells, we first examined the cells by transmission electron microscopy. Compared with the WT group, APEC OMVs-treated cells showed damaged mitochondria enclosed by autophagic structures and associated with lysosomal structures (Fig. 3A, B). Furthermore, indirect immunofluorescence revealed that APEC OMVs promoted the colocalisation of mitochondria with LC3 and LAMP1; however, this colocalisation was significantly reduced following treatment with the mitophagy inhibitor Mdivi-1 (Fig. 3C, D). To further corroborate these findings, we assessed protein expression by Western blotting and mtDNA copy number by qPCR in HD11 cells. The results showed that, compared with the WT control, the conversion of LC3-Ⅰ to LC3-ⅠI was significantly reduced following infection with Δ*ypjA* (*P* < 0.01); the extent of degradation of the mitochondrial proteins TIMM23 and TOM20 was also significantly reduced (*P* < 0.01) (Fig. 4A). Following 6 h of treatment with APEC OMVs, the degradation of the mitochondrial marker proteins TOM20 and TIMM23 was pronounced (*P* < 0.001), and the conversion of LC3-Ⅰ to LC3-ⅠI increased significantly (*P* < 0.01) (Fig. 4B). Concurrently, following APEC infection, intracellular mtDNA levels decreased significantly (*P* < 0.01), with the decline being markedly greater in the WT group than in the Δ*ypjA* group (*P* < 0.05). Similarly, the mtDNA content also decreased significantly after 6 h of treatment with APEC OMVs (*P* < 0.01) (Fig. 4C). Notably, treatment with Mdivi-1 effectively reversed the APEC OMVs-induced degradation of TOM20 and TIMM23 (*P* < 0.01) (Fig. 4D, E).

### *APEC Promotes systemic infection by inducing mitophagy via the secretion of OMVs*

**Fig. 5:**
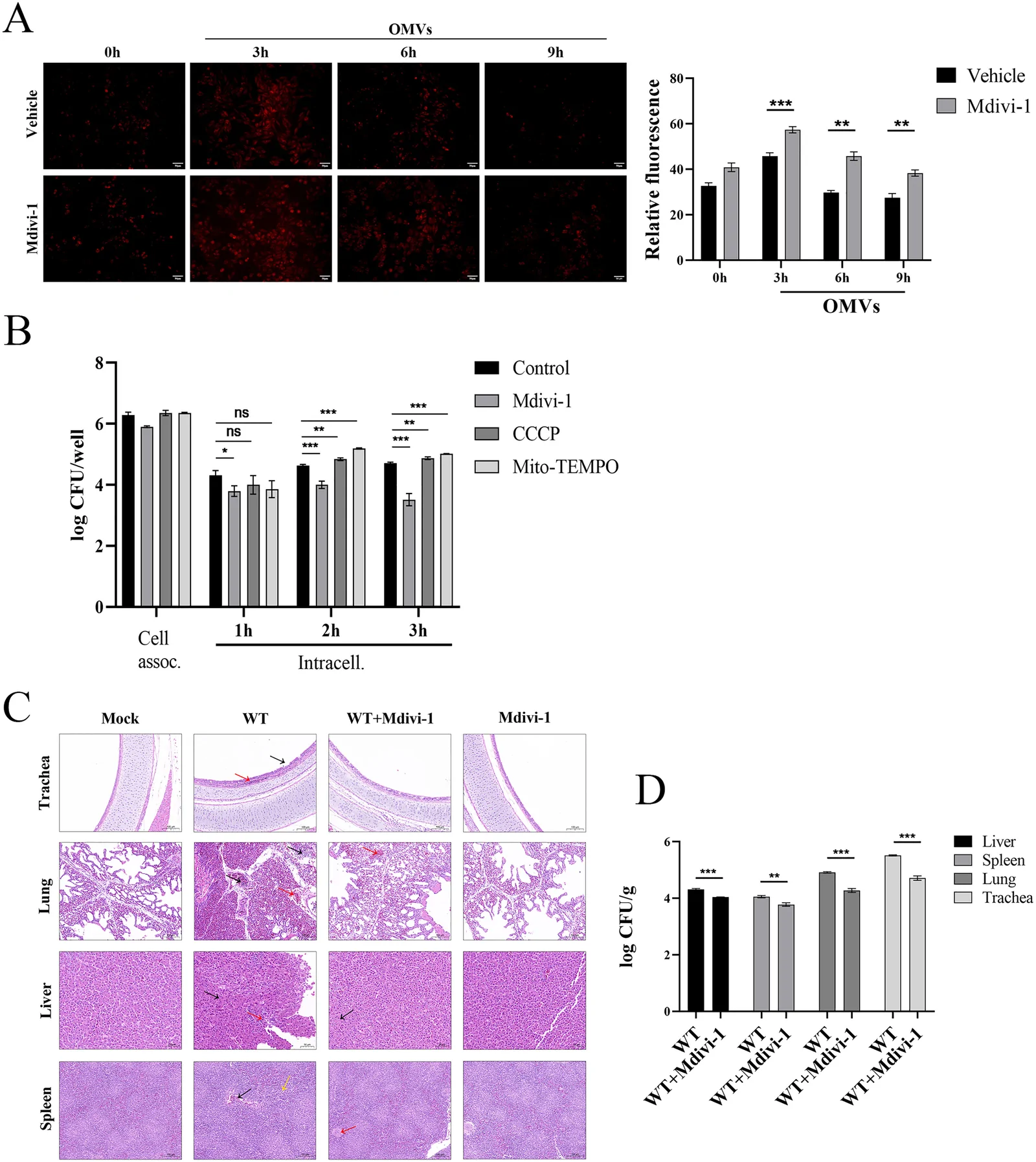
**Mitophagy induced by OMVs secreted by APEC promotes systemic infection.** (A) Dynamic changes in ROS levels in HD11 cells following APEC OMVs treatment. ROS levels initially increased and then gradually decreased. Mdivi-1 treatment significantly delayed the decline in ROS levels. The fluorescence signals were quantified using ImageJ. Data represent one of three independent experiments. Scale bar = 50 μm. (B) Intracellular survival of WT following infection of HD11 cells with MOI = 100. Mdivi-1 significantly reduced the intracellular survival of WT, whilst Mito-TEMPO and CCCP significantly increased it. (n = 3). (C) Six days after infection of chicks (1 × 10⁹ CFU per chick) with WT, in the absence or presence of Mdivi-1 (20 μM, 2 h), tracheal, lung, spleen and liver tissues were harvested for H&E staining. Trachea: deformation and desquamation of epithelial cells with broken cilia (black arrow); inflammatory cell infiltration in the lamina propria (red arrow); Lung: inflammatory cell infiltration in the pulmonary stroma (black arrow); marked thickening of the pulmonary septa; significant reduction in airspace area; marked dilation and congestion of capillaries (red arrow); Liver: inflammatory cell infiltration (red arrow); hepatocytes with blurred borders and markedly abnormal morphology (black arrow); Spleen: dilated red pulp accompanied by haemorrhage and diffuse inflammatory cell infiltration (black arrow); reduced lymphocyte count (yellow arrow); and markedly thickened vessel walls (red arrow). Scale bar = 50 µm or 100 µm. (D) Bacterial load in the trachea, lungs, spleen and liver 6 days after infection of WT chicks (1 × 10⁹ CFU/bird) in the absence or presence of Mdivi-1. Following treatment with Mdivi-1, the bacterial load in the trachea, lungs, spleen and liver was significantly reduced. (n = 3). n represents three biological replicates; data points correspond to independent culture systems; bar graphs show means ± standard errors; two-way ANOVA with Sidak correction (* *P* < 0.05, ** *P* < 0.01, *** *P* < 0.001).

Mitochondria in eukaryotic cells can detect pathogen invasion and suppress pathogen invasion by producing large amounts of ROS; however, excessive mitophagy leads to a reduction in mitochondrial numbers, thereby limiting ROS production. Because mitophagy is closely associated with mitochondrial homeostasis and ROS levels, we further examined the dynamic changes in ROS levels in HD11 cells treated with APEC OMVs for 0, 3, 6, and 9 h. Compared with the control group (0 h), ROS levels were significantly increased after 3 h of APEC OMVs treatment. ROS levels gradually decreased after 6 h of treatment. To further assess the relationship between mitophagy and ROS changes, we used Mdivi-1 for pharmacological intervention. Mdivi-1 markedly slowed the decline in ROS levels (*P* < 0.01) (Fig. 5A). These results suggest that mitophagy may contribute to the dynamic changes in ROS induced by APEC OMVs.

We then investigated whether the reduction in ROS caused by mitophagy could affect the survival of APEC within HD11 cells, The results show that Mdivi-1 pretreatment of HD11 cells significantly reduced APEC survival as early as 1 h post-internalisation (*P* < 0.05), whereas induction of mitophagy with CCCP or treatment with the ROS quencher Mito-TEMPO enhanced intracellular survival at 2 h post-internalisation (*P* < 0.01) (Fig. 5B). Next, we used APEC81 WT to validate the infection in chicks *in vivo*. Histopathological examination revealed that the histopathological changes in the WT group were similar to those described previously (Fig. 1D), whilst neither the control group (PBS-treated) nor the Mdivi-1 group (receiving continuous injections of Mdivi-1) exhibited obvious pathological damage in any tissue. Compared with the group infected with WT alone, the tracheal mucosal epithelium of infected chicks in the WT+Mdivi-1 group was arranged in a relatively orderly and continuous manner, with no obvious inflammatory cell infiltration observed; there were only a small number of inflammatory cells in the pulmonary stroma, which showed no significant thickening, and the lumens remained relatively unobstructed; the margins of the hepatocytes were relatively well-defined, with only a small number of inflammatory cells present; in the spleen, the boundaries between the white pulp and red pulp were relatively well-defined, with mild haemorrhage in the red pulp and a normal lymphocyte count (Fig. 5C). We subsequently analysed the bacterial load in the various tissues of the chicks; the bacterial load in all tissues of the chicks in the WT+Mdivi-1 group was significantly lower than that in the WT group (*P* < 0.01) (*P* < 0.001) (Fig. 5D).

### *APEC OMVs regulate mitophagy via MSTRG.11745.1*

**Fig. 6:**
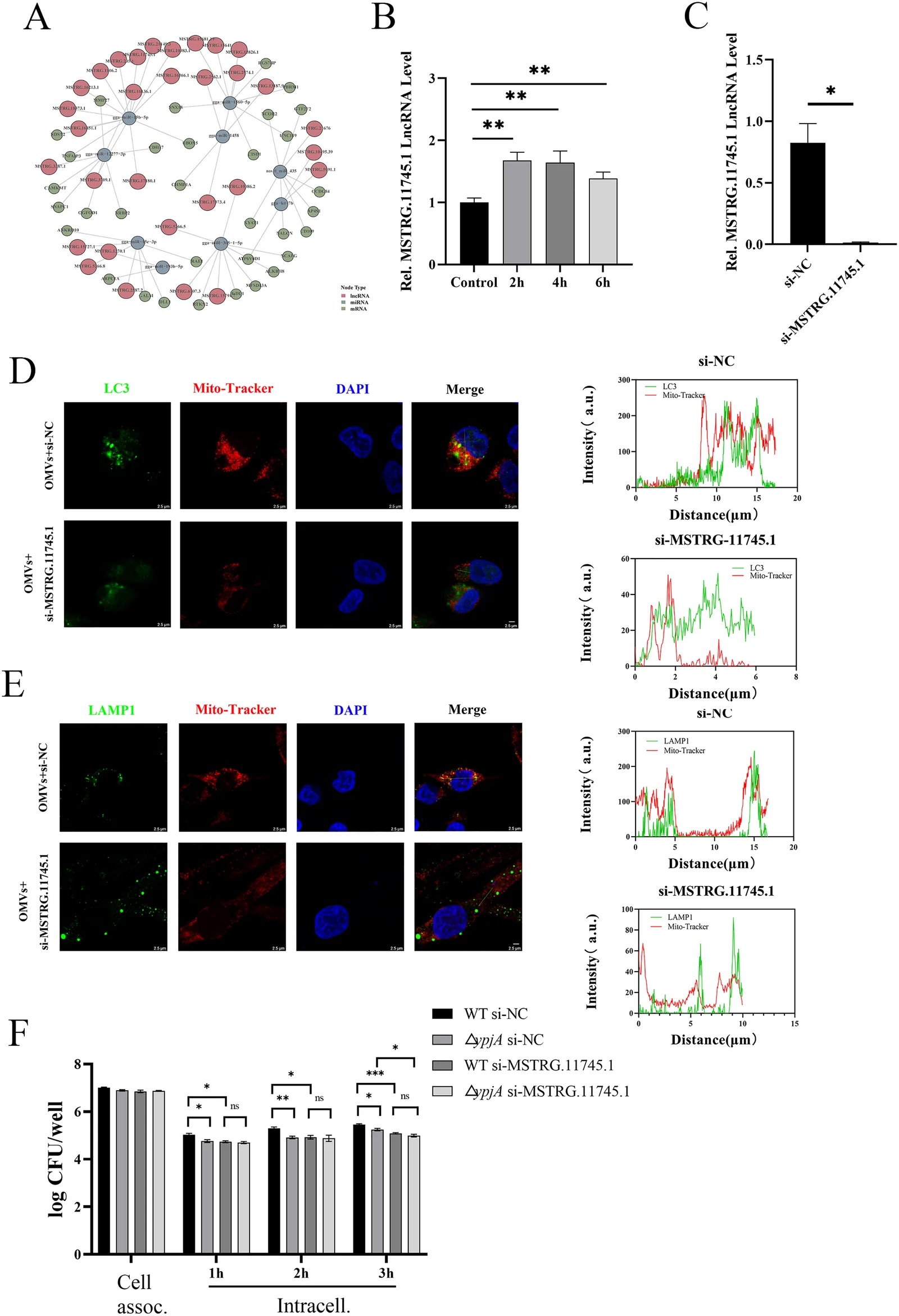
**MSTRG.11745.1 expression correlates positively with mitophagy levels under APEC OMVs treatment.** (A) ceRNA regulatory network in HD11 cells following treatment with APEC OMVs. The ceRNA regulatory network was constructed based on transcriptomic sequencing, combined with predictions of lncRNA–miRNA and miRNA–mRNA targeting relationships. Red nodes represent lncRNAs, blue nodes represent miRNAs, green nodes represent mRNAs, and the connecting lines indicate predicted regulatory relationships. (B) APEC OMVs significantly increased the expression level of MSTRG.11745.1 in HD11 cells. (n = 3). (C) The knockdown efficiency of MSTRG.11745.1 was assessed by qPCR. (n = 3). (D) Knockdown of MSTRG.11745.1 significantly reduced the colocalization of mitochondria with LC3 in APEC OMVs-treated HD11 cells. Data represent one of three independent experiments. Scale bar = 2.5 μm. Co-localisation scatter plots were generated using ImageJ. (E) Knockdown of MSTRG.11745.1 significantly reduced the colocalization of mitochondria with LAMP1 in APEC OMVs-treated HD11 cells. Data represent one of three independent experiments. Scale bar = 2.5 μm. Co-localisation scatter plots were generated using ImageJ. (F) Knockdown of MSTRG.11745.1 abolished the difference in intracellular survival between WT and Δ*ypjA* strains in HD11 cells. (n = 3). n represents three biological replicates; data points correspond to independent culture systems; bar graphs show means ± standard errors; analysed using Student’s t-test. (* *P* < 0.05, ** *P* < 0.01, *** *P* < 0.001).

To identify the upstream lncRNA through which APEC OMVs regulate mitophagy in host cells (Fig. S2), we first performed transcriptomic sequencing (RNA-seq) analysis and constructed a ceRNA network diagram (Fig. 6A). Experimental validation revealed that, following treatment with APEC OMVs, the expression level of a lncRNA named MSTRG.11745.1 was significantly up-regulated (*P* < 0.01) (Fig. 6B). Further target gene prediction and co-expression analysis indicated that the potential target genes of MSTRG.11745.1 were heavily enriched in pathways related to mitophagy. We therefore hypothesised that MSTRG.11745.1 might be a key regulatory factor mediating APEC OMVs-induced mitophagy. Subsequently, to further validate the regulatory role of MSTRG.11745.1 in mitophagy, we performed MSTRG.11745.1 knockdown (Fig. 6C). Using laser confocal microscopy, we observed that after 6 h of co-culture of APEC OMVs with HD11 cells, the co-localisation of mitochondria with LC3 and LAMP1 was significantly reduced in the knockdown group compared with the negative control group (Fig. 6D, E). To further investigate the effect of MSTRG.11745.1 on APEC cell viability, MSTRG.11745.1 was similarly silenced. Knockdown of MSTRG.11745.1 significantly decreased the intracellular survival of the WT strain, with significant reductions detected at 1 and 2 h (*P* < 0.05) and a greater reduction at 3 h (*P* < 0.01). In the si-NC group, WT exhibited significantly higher intracellular survival than Δ*ypjA* at 1, 2, and 3 h. Notably, this difference was abolished following MSTRG.11745.1 knockdown, as no significant difference in intracellular survival was detected between WT and Δ*ypjA* at any time point (1, 2, or 3 h) (*P* > 0.05) (Fig. 6F).

### *MSTRG.11745.1 positively regulates TNFAIP3-induced mitophagy*

**Fig. 7:**
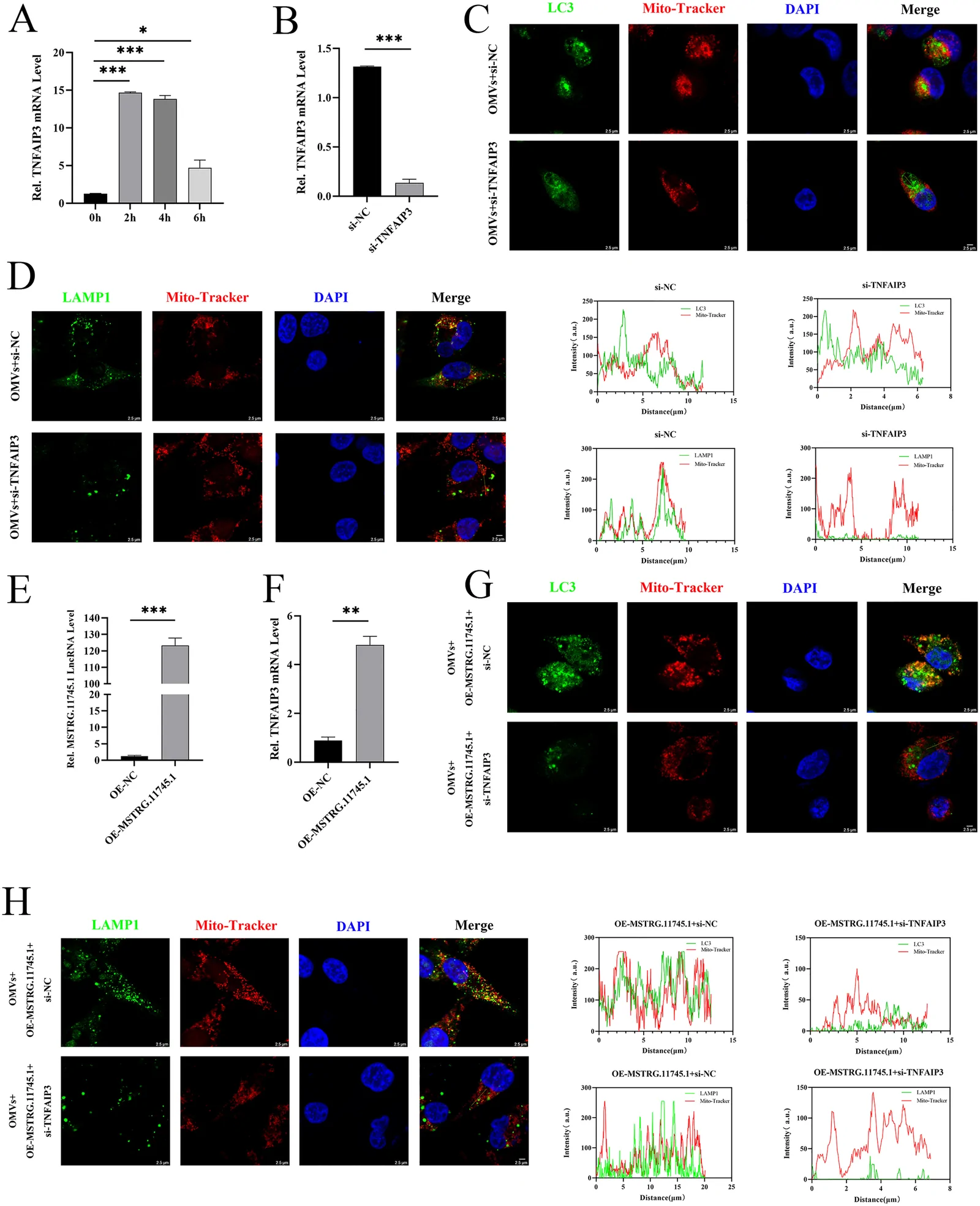
**MSTRG.11745.1 upregulates *TNFAIP3*-induced mitophagy.** (A) APEC OMVs treatment significantly upregulated *TNFAIP3* mRNA expression in HD11 cells. (n = 3). (B) The efficiency of *TNFAIP3* knockdown was assessed by qPCR. (n = 3). (C) Following *TNFAIP3* knockdown, the colocalisation of mitochondria with LC3 in HD11 cells treated with APEC OMVs was significantly reduced. Data represent one of three independent experiments. Scale bar = 2.5 μm. Co-localisation scatter plots were generated using ImageJ. (D) Following *TNFAIP3* knockdown, the colocalisation of mitochondria with LAMP1 in HD11 cells treated with APEC OMVs was significantly reduced. Data represent one of three independent experiments. Scale bar = 2.5 μm. Co-localisation scatter plots were generated using ImageJ. (E) qPCR analysis of the overexpression efficiency of MSTRG.11745.1. (n = 3). (F) Overexpression of MSTRG.11745.1 significantly upregulates the mRNA levels of *TNFAIP3*. (n = 3). (G) MSTRG.11745.1 overexpression enhances the APEC OMVs-induced colocalisation of mitochondria with LC3, whilst *TNFAIP3* knockdown attenuates this effect. Data represent one of three independent experiments. Scale bar = 2.5 μm. Co-localisation scatter plots were generated using ImageJ. (H) MSTRG.11745.1 overexpression enhances the APEC OMVs-induced colocalisation of mitochondria with LAMP1, whilst *TNFAIP3* knockdown attenuates this effect. Data represent one of three independent experiments. Scale bar = 2.5 μm. Co-localisation scatter plots were generated using ImageJ. n represents three biological replicates; data points correspond to independent culture systems; bar graphs show mean ± standard error; analysis was performed using Student’s t-test (* *P* < 0.05, ** *P* < 0.01, *** *P* < 0.001).

To investigate the downstream genes regulated by MSTRG.11745.1, we screened for mRNAs co-expressed with MSTRG.11745.1: *TNFAIP3*. HD11 cells were treated with APEC OMVs for 2, 4, and 6 h to assess *TNFAIP3* expression. Compared with the control group, *TNFAIP3* expression was significantly increased at 6 h after OMVs treatment (*P* < 0.05) (Fig. 7A). We then investigated the relationship between *TNFAIP3* and mitophagy. Following siRNA interference against *TNFAIP3* (Fig. 7B), laser confocal microscopy revealed that, after 6 h of co-culture of APEC OMVs with HD11 cells, the colocalisation of mitochondria with LC3 and LAMP1 was markedly reduced in the siRNA-treated group compared with the control group (Fig. 7C, D). Subsequently, we overexpressed MSTRG.11745.1 (Fig. 7E) and found that *TNFAIP3* was up-regulated when co-expressed with it (*P* < 0.01) (Fig. 7F). To further verify whether *TNFAIP3* is a key downstream target gene regulated by MSTRG.11745.1, we conducted a rescue experiment in HD11 cells. MSTRG.11745.1 was first overexpressed, followed by *TNFAIP3* knockdown and APEC OMVs treatment. Mitochondrial–LC3 and mitochondrial–LAMP1 colocalization was then assessed by confocal microscopy.

The results show that, under OMVs treatment conditions, compared with the overexpression of MSTRG.11745.1 alone, the simultaneous overexpression of MSTRG.11745.1 and siRNA targeting *TNFAIP3* significantly reversed the increase in mitochondrial colocalisation with LC3 and LAMP1 caused by MSTRG.11745.1 overexpression (Fig. 7G, H).

### *MSTRG.11745.1 regulates mitophagy through the gga-miR-15b-5p/TNFAIP3 axis*

**Fig. 8:**
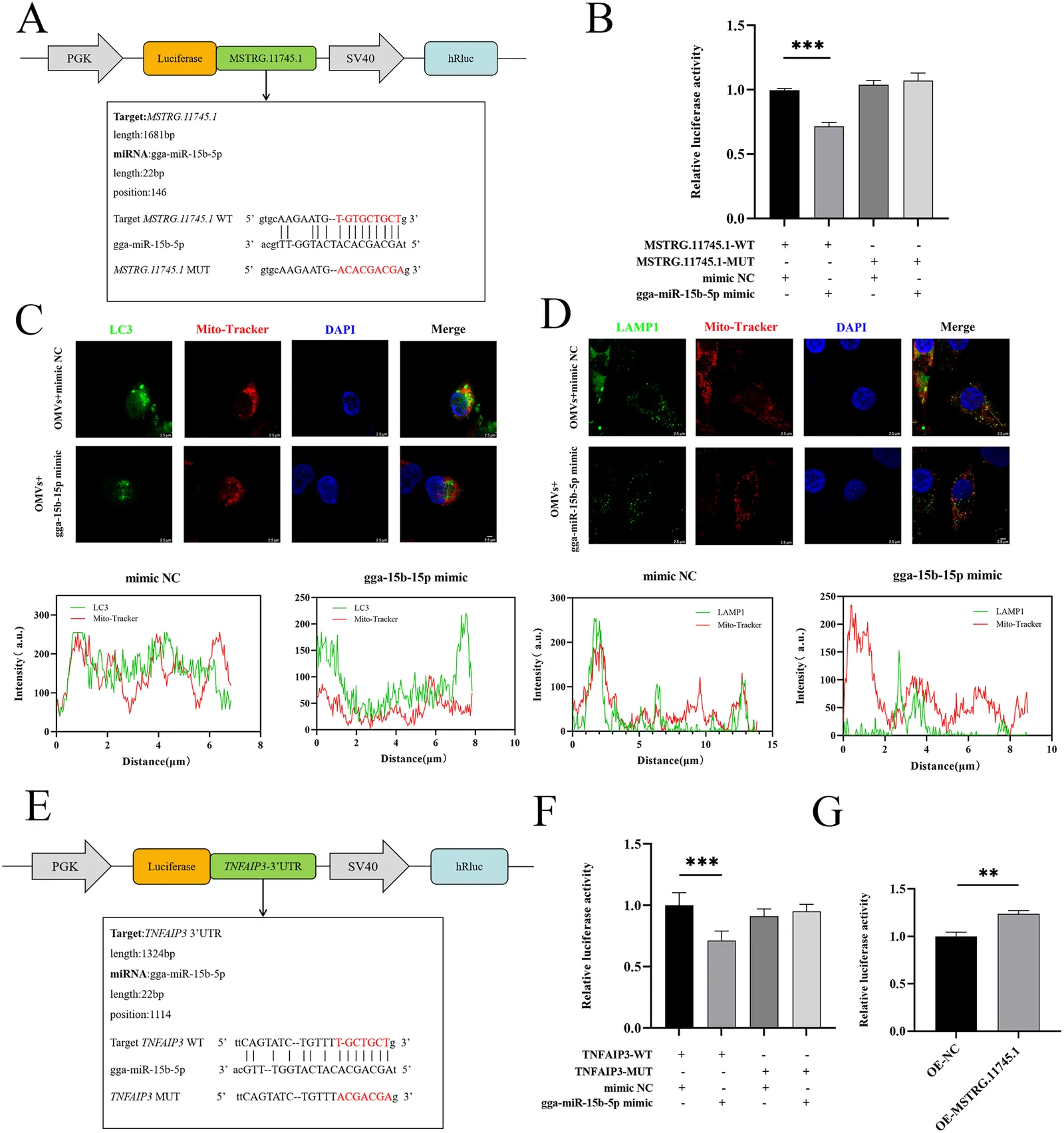
**MSTRG.11745.1 regulates mitophagy by targeting the gga-miR-15b-5p/*TNFAIP3* axis.** (A) Predicted binding sites of gga-miR-15b-5p and MSTRG.11745.1, and schematic diagrams of wild-type and mutant pmirGLO-MSTRG.11745.1 constructs. (B) Dual-luciferase reporter assay. MSTRG.11745.1 interacts with gga-miR-15b-5p at the predicted binding site. (n = 3). (C) Following overexpression of gga-miR-15b-5p, the colocalisation of mitochondria with LC3 in HD11 cells treated with APEC OMVs was significantly reduced. Data represent one of three independent experiments. Scale bar = 2.5 μm. Co-localisation scatter plots were generated using ImageJ. (D) Following overexpression of gga-miR-15b-5p, the colocalisation of mitochondria with LAMP1 in HD11 cells treated with APEC OMVs was significantly reduced. Data represent one of three independent experiments. Scale bar = 2.5 μm. Co-localisation scatter plots were generated using ImageJ. (E) Schematic diagram showing the predicted binding site of gga-miR-15b-5p to the *TNFAIP3* 3′UTR, and the wild-type and mutant pmirGLO-*TNFAIP3* 3′UTR constructs. (F) Dual-luciferase reporter assay. gga-miR-15b-5p interacts with the 3′UTR of *TNFAIP3* at the predicted binding site. (n = 3). (G) Overexpression of MSTRG.11745.1 reduces the efficiency with which gga-miR-15b-5p binds to *TNFAIP3*. (n = 3). n represents three biological replicates; data points correspond to independent culture systems; bar graphs show mean ± standard error; analysed using Student’s t-test (** *P* < 0.01, *** *P* < 0.001).

To investigate whether MSTRG.11745.1 acts as a miRNA sponge via the competitive endogenous RNA mechanism, we first predicted potential binding sites between MSTRG.11745.1 and gga-miR-15b-5p through bioinformatic analysis (Fig. 8A). Subsequently, we validated their interaction using a dual-luciferase reporter assay. The results showed that the fluorescence intensity of the dual-luciferase reporter was significantly reduced upon co-transfection of MSTRG.11745.1-WT and gga-miR-15b-5p mimic (*P* < 0.001), whereas the co-transfection of MSTRG.11745.1-MUT with the gga-miR-15b-5p mimic showed no significant change in reporter fluorescence intensity (Fig. 8B), indicating a direct binding interaction between MSTRG.11745.1 and gga-miR-15b-5p. Subsequently, to investigate whether gga-miR-15b-5p affects mitophagy, we performed overexpression experiments using cells transfected with the gga-miR-15b-5p mimic and assessed mitophagy levels via immunofluorescence colocalisation analysis. The results showed that, compared with the negative control (NC) group, overexpression of gga-miR-15b-5p significantly attenuated the colocalisation signals between mitochondria and LC3 as well as LAMP1 (Fig. 8C, D) . To further investigate whether gga-miR-15b-5p exerts its inhibitory effect by binding to the 3′ UTR region of *TNFAIP3*, Similarly, we identified potential binding sites between the two using bioinformatics (Fig. 8E). We subsequently validated this using a dual-luciferase reporter assay; the results indicated that the luciferase activity was significantly reduced in the co-transfection of *TNFAIP3*-WT and gga-miR-15b-5p mimic (*P* < 0.001), whereas there was no significant change in luciferase activity in the co-transfection of *TNFAIP3*-MUT and gga-miR-15b-5p mimic (Fig. 8F). Finally, to verify whether MSTRG.11745.1 alleviates the inhibitory effect of gga-miR-15b-5p on downstream *TNFAIP3* by binding to it, we overexpressed MSTRG.11745.1 following co-transfection of *TNFAIP3*-WT and the gga-miR-15b-5p mimic. The dual-luciferase reporter assay results showed that overexpression of MSTRG.11745.1 significantly reversed the reduction in fluorescence intensity (*P* < 0.01), indicating that it restored the suppressed luciferase activity (Fig. 8G).

## Discussion

Persistent infection by pathogenic bacteria is highly dependent on their ability to evade host immune clearance and survive intracellularly (Zhuge et al., 2018; Yu et al., 2025). In the case of APEC, the establishment of systemic infection involves the coordination of multiple virulence mechanisms, including adhesion, invasion, intracellular survival and systemic dissemination (Khairullah et al., 2024). Macrophages, as the first line of defence against bacterial infection, are pivotal in the host–pathogen interaction (Mellata et al., 2003; Zhuge et al., 2019). To evade host immune killing and achieve persistent infection, APEC is capable of activating the HD11 cellular immune signalling pathway, thereby causing cellular damage (Wang et al., 2023). OMVs act as “transport vehicles” for pathogens to deliver virulence factors. They can be internalized by macrophages and exert pathogenic effects. OMVs also play important roles in the pathogenic processes of APEC, UPEC, and *P. aeruginosa* (Deo et al., 2020; Li et al., 2024). In this study, we found that the intracellular survival capacity and the extent of pathological damage in the OMVs-secretion-deficient strain were both significantly lower than those of the wild-type strain. This is consistent with the findings from our previous study, which demonstrated that APEC OMVs can promote systemic infection and intracellular survival (Cui et al., 2026). These results indicate that APEC OMVs induce persistent systemic infection by enhancing *in vivo* colonisation and intracellular survival. In response to pathogen invasion, the host often initiates cellular autophagy to eliminate pathogens. NOD1, as an intracellular immune recognition receptor, can be activated by recognising peptidoglycan (**PG**) fragments, thereby triggering autophagy and inflammatory responses (Lenz et al., 2017). This study found that upon knockout of the *ldcA* gene in APEC, the OMVs secreted by the bacterium significantly reduced overall autophagy levels after inhibiting NOD1-dependent autophagy. These findings suggest that NOD1 signaling may be involved in APEC OMVs-induced autophagy but is unlikely to be the sole regulatory mechanism. Other NOD1-independent pathways may also contribute to this process. These results are consistent with recent findings on OMVs secreted by *ldcA*-deficient *Neisseria gonorrhoeae* (Gao et al., 2024). Consequently, OMVs and the virulence factors they carry exert complex regulatory effects on the host’s immune defences.

Mitochondria are the central organelles involved in energy metabolism and signal regulation in eukaryotic cells, and the integrity of their structure and function is directly linked to cellular homeostasis (Rotimi et al., 2024). In macrophages, mitochondria respond rapidly to invading pathogens. They generate large amounts of mitochondrial reactive oxygen species (**mtROS**) through the electron transport chain. mtROS can kill intracellular bacteria and activate pro-inflammatory pathways (Ellzey et al., 2023). Our study showed that APEC OMVs are internalized by HD11 cells and target mitochondria. Bacterial OMVs can enter host cells through endocytosis and release virulence factors intracellularly (Bielaszewska et al., 2017). Similarly, *Neisseria gonorrhoeae* OMVs have been reported to interact with mitochondria and deliver PorB, thereby inducing mitophagy (Gao et al., 2024). We therefore speculate that APEC OMVs may deliver virulence factors to mitochondria through endosomal transport or endosome–mitochondria contacts, leading to mitochondrial dysfunction. However, the underlying mechanisms require further investigation. Our study also showed that APEC OMVs disrupted the mitochondrial network structure. They caused a transient increase in ROS levels and a gradual decrease in mitochondrial membrane potential (**ΔΨm**). The transient increase in ROS may reflect an acute stress response during the early stage of mitochondrial damage. Similarly, previous studies have shown that the APEC-secreted Hcp2a protein can target mitochondria in DF-1 cells. It reduces mitochondrial membrane potential and increases ROS levels (Lu et al., 2024). Notably, a defect in vesicular secretion significantly attenuated this impairment of membrane potential. This suggests that APEC relies on OMVs to disrupt mitochondrial function, thereby compromising the host cell’s ability to kill the pathogen. To evade mitochondria-mediated immune clearance, pathogens can disrupt host mitochondrial function by secreting OMVs. They may also modulate mitophagy and hijack host defense responses to establish infection.

In this study, we provide direct evidence that APEC can induce mitophagy in HD11 cells through the secretion of OMVs. Previous studies have shown that bacterial OMVs can contribute to pathogen–host interactions by damaging host mitochondria and inducing mitophagy. (Gao et al., 2024). In fact, using mitophagy to reduce intracellular ROS is a common strategy employed by various pathogens to promote their persistent survival.(Xu et al., 2023; Yang et al., 2023; Gao and van der Veen, 2024; Sun et al., 2025). In the present study, intracellular ROS levels gradually decreased in macrophages after 6 h of treatment. This decrease was significantly attenuated by treatment with a mitophagy inhibitor. The degradation of mitochondrial membrane proteins was also reduced. This suggests that APEC clears damaged mitochondria precisely through OMVs-mediated autophagy, thereby reducing intracellular ROS accumulation. However, our results only indicate an association between mitophagy and ROS changes. ROS levels may also be influenced by other regulatory pathways, such as inflammatory signaling and antioxidant responses. Our further studies showed that inhibition of mitophagy significantly reduced the intracellular survival of APEC in HD11 macrophages. It also reduced the bacterial load in various tissues in the chick infection model. In addition, inhibition of mitophagy significantly alleviated APEC-associated pathological damage. This finding is consistent with previous studies showing that mitophagy contributes to the regulation of intracellular pathogen survival (Song et al., 2022). Our findings suggest that the targeted inhibition of mitophagy may be an effective strategy for suppressing persistent APEC infection. A limitation of this study is that the specific components of OMVs responsible for inducing this regulation of mitophagy remain unclear. Future research should employ systematic screening and validation experiments to elucidate the role of key virulence factors within OMVs in this mechanism.

Conventional studies of mitophagy regulation have typically focused on signalling cascades at the protein level, such as the PINK1-PRKN pathway and the mediation of related autophagy receptors (Rotimi et al., 2024). However, a growing body of research indicates that, beyond traditional protein-based regulatory networks, non-coding RNAs (particularly lncRNAs) play an equally critical regulatory role in pathogen-induced host immune responses (Jiang et al., 2021; Xu et al., 2021; Ning et al., 2024; Han et al., 2025). In studies of Mycobacterium tuberculosis (M. tuberculosis) infection, LncRNA-CFTBS enhances the survival of the pathogen within macrophages by regulating ferroptosis. In this study, a novel lncRNA—MSTRG.11745.1—was identified in HD11 cells treated with APEC OMVs; it exerts a positive regulatory effect on mitophagy, and inhibition of its expression suppresses the intracellular survival of APEC and eliminates the differences in intracellular survival induced by OMVs. LncRNAs regulate gene expression by acting as miRNA sponges, competitive endogenous RNAs, decoys or scaffolds (Tang et al., 2019). By integrating transcriptomic sequencing data with miRNA predictions, We have established that MSTRG.11745.1 acts as a competitive endogenous RNA that interacts with gga-miR-15b-5p and regulates its availability to the target gene *TNFAIP3*, and demonstrated that gga-miR-15b-5p inhibits mitophagy. However, the effects of MSTRG.11745.1 knockdown on the expression level of gga-miR-15b-5p were not examined in this study. Further studies using RNA pull-down and related approaches will be conducted to investigate the direct interaction between MSTRG.11745.1 and gga-miR-15b-5p. Similarly, in M. tuberculosis, miR-1301-3p and miR-5194 have been shown to inhibit macrophage autophagy (Qu et al., 2023). More importantly, our experiments demonstrated that MSTRG.11745.1 promotes APEC OMVs-induced mitophagy in a *TNFAIP3*-dependent manner. These findings suggest that pathogens may promote mitophagy by manipulating the host non-coding RNA network. This post-transcriptional regulatory strategy may reshape the host immune microenvironment and contribute to persistent infection by various intracellular pathogens. This study has several limitations. First, we mainly used knockdown experiments to investigate the regulatory roles of MSTRG.11745.1 and *TNFAIP3* in mitophagy. The effects of their overexpression on this process have not been further validated. Second, functional validation of the MSTRG.11745.1/gga-miR-15b-5p/*TNFAIP3* axis was mainly performed in the *in vitro* HD11 cell model. Its roles in mitophagy and APEC infection *in vivo* remain to be determined. The underlying molecular mechanisms also require further investigation in animal models. Because ceRNA networks involve complex interactions among multiple RNA molecules, future studies should investigate additional ceRNA axes involved in APEC infection. This may help to further clarify the complexity of APEC infection and its immune evasion strategies.

Beyond its canonical anti-inflammatory function, *TNFAIP3* has also been implicated in autophagy and mitochondrial quality control. Previous studies suggest that *TNFAIP3* can promote autophagy and mitophagy through pathways involving mTOR and BNIP3, and may help maintain mitochondrial homeostasis by reducing mitochondrial ROS levels (Shembade et al., 2010; Matsuzawa et al., 2015; Peng et al., 2022; White et al., 2023). Consistent with these findings, our results showed that *TNFAIP3* positively regulates APEC OMVs-induced mitophagy. We speculate that *TNFAIP3* may facilitate the clearance of damaged mitochondria through its ubiquitin-editing activity. However, the precise mechanism and the involvement of pathways such as mTOR, BNIP3, and PINK1/PRKN require further investigation.

**Fig. 9:**
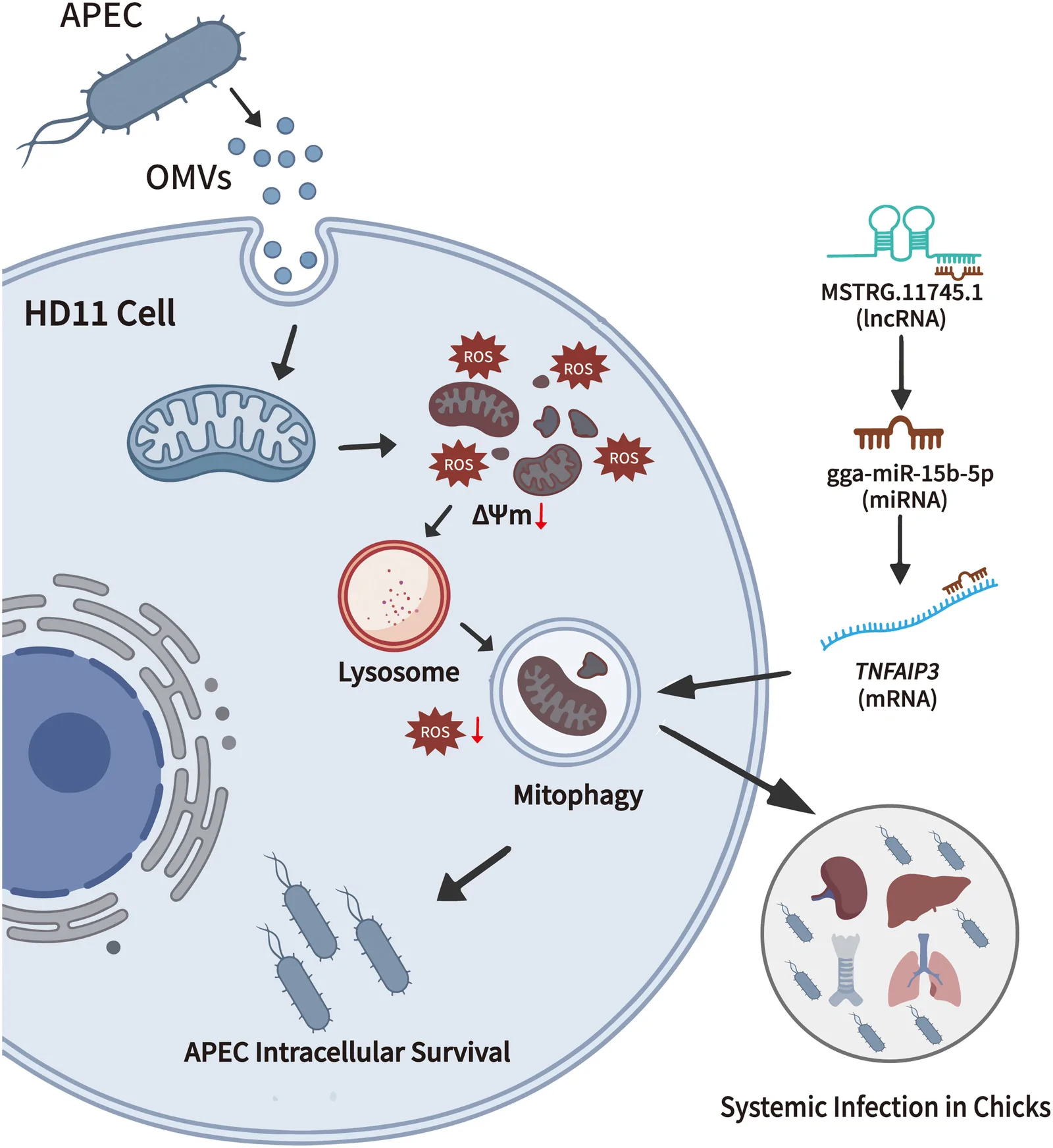
**A model showing that OMVs secreted by APEC regulate mitophagy via the MSTRG.11745.1/gga-miR-15b-5p/*TNFAIP3* axis, thereby promoting their survival within macrophages and systemic infection.** Following uptake by HD11 cells, APEC-derived OMVs target the mitochondria and cause mitochondrial damage. The damaged mitochondria are subsequently removed through mitophagy mediated by the MSTRG.11745.1/gga-miR-15b-5p/*TNFAIP3* axis. This process reduces intracellular ROS accumulation and promotes APEC survival in HD11 cells. It may also facilitate bacterial immune evasion and contribute to systemic infection.

In summary, our results indicate that APEC-derived OMVs target mitochondria in HD11 cells and cause mitochondrial damage. This damage induces mitophagy through the MSTRG.11745.1/gga-miR-15b-5p/*TNFAIP3* axis. Mitophagy reduces intracellular ROS levels and promotes APEC survival in HD11 cells. It may also contribute to systemic infection (Fig. 9). This study provides a new perspective on the role of OMVs in mediating APEC immune evasion and lays a theoretical foundation for further exploration of immunomodulatory strategies against APEC disease.

## Author Contribution

JT, ZYC, and XJS participated in the conception and design of the study. JYG, ZL, and TTC performed the experiments. YS, ZYW, and JMS analyzed the data. JYG wrote the manuscript. All authors were involved in revising the manuscript, reading it, and approving the submitted version.

## Funding

This study was supported by the 10.13039/501100001809National Natural Science Foundation of China (grant nos. 32573322), 10.13039/501100012166National Key Research and Development Program of China (2023YFD1702102-11), Hefei Municipal Natural Science Foundation (HZR2427), and Research Funds of Joint Research Center for Food Nutrition and Health of IHM (2024SJY04, 2023SJY01). The funding bodies had no role in the design of the study and collection, analysis, and interpretation of data and in writing the manuscript.

## Ethics declaration

This study was conducted in accordance with the ARRIVE (Animal Research: Reporting of In Vivo Experiments) guidelines. This study was approved by the Animal Experimentation Ethics Committee of Anhui Agricultural University. (Approval No. KJLLXM2025104).

## Disclosures

The authors declare that they have no competing interests.

## Appendix Supplementary materials

Fig. S1: **Transmission electron microscopy analysis of mitochondrial damage in HD11 cells treated with APEC OMVs.** (A) Representative transmission electron microscopy images of mitochondria in HD11 cells treated with DMEM (control). (B) Representative transmission electron microscopy images of mitochondria in HD11 cells treated with APEC OMVs. Scale bar = 5 μm.

Fig. S2**: GO enrichment and KEGG pathway enrichment analysis of target genes of differentially expressed lncRNAs in OMVs-treated HD11 cells.** (A) GO enrichment analysis of the target genes of differentially expressed lncRNAs. (B) KEGG pathway enrichment analysis of the target genes of differentially expressed lncRNAs.

## Footnotes

Supplementary material associated with this article can be found, in the online version, at doi:10.1016/j.psj.2026.107755. Fig. S1: **Transmission electron microscopy analysis of mitochondrial damage in HD11 cells treated with APEC OMVs.** (A) Representative transmission electron microscopy images of mitochondria in HD11 cells treated with DMEM (control). (B) Representative transmission electron microscopy images of mitochondria in HD11 cells treated with APEC OMVs. Scale bar = 5 μm. Fig. S2**: GO enrichment and KEGG pathway enrichment analysis of target genes of differentially expressed lncRNAs in OMVs-treated HD11 cells.** (A) GO enrichment analysis of the target genes of differentially expressed lncRNAs. (B) KEGG pathway enrichment analysis of the target genes of differentially expressed lncRNAs.

## References

[bib1] Antão E.-M., Ewers C., Gürlebeck D., Preisinger R., Homeier T., Li G., Wieler L.H. (2009). Signature-tagged mutagenesis in a chicken infection model leads to the identification of a novel avian pathogenic *Escherichia coli* fimbrial adhesin. PLoS. One.

[bib2] Avila-Calderón E.D., Del Ruiz-Palma M.S, Aguilera-Arreola M.G., Velázquez-Guadarrama N., Ruiz E.A., Gomez-Lunar Z., Witonsky S., Contreras-Rodríguez A. (2021). Outer membrane vesicles of gram-negative bacteria: an outlook on biogenesis. Front. Microbiol..

[bib4] Bielaszewska M., Rüter C., Kunsmann L., Greune L., Bauwens A., Zhang W., Kuczius T., Kim K.S., Mellmann A., Schmidt M.A., Karch H. (2013). Enterohemorrhagic *Escherichia coli* hemolysin employs outer membrane vesicles to target mitochondria and cause endothelial and epithelial apoptosis. PLoS. Pathog..

[bib3] Bielaszewska M., Rüter C., Bauwens A., Greune L., Jarosch K.-A., Steil D., Zhang W., He X., Lloubes R., Fruth A., Kim K.S., Schmidt M.A., Dobrindt U., Mellmann A., Karch H. (2017). Host cell interactions of outer membrane vesicle-associated virulence factors of enterohemorrhagic *Escherichia coli* O157: intracellular delivery, trafficking and mechanisms of cell injury. PLoS. Pathog..

[bib5] Cui T., Li Z., Gao J., Miao Z., Li F., Shao Y., Wang Z., Sun J., Song X., Qi K., Tu J. (2026). Outer membrane vesicles secreted by avian pathogenic *Escherichia coli* promote its survival within macrophages and systemic infection by inducing endoplasmic reticulum stress-mediated autophagy flux blockade. Vet. Res..

[bib6] Datsenko K.A., Wanner B.L. (2000). One-step inactivation of chromosomal genes in *Escherichia coli* K-12 using PCR products. Proc. Natl. Acad. Sci. USA.

[bib8] Deo P., Chow S.H., Hay I.D., Kleifeld O., Costin A., Elgass K.D., Jiang J.-H., Ramm G., Gabriel K., Dougan G., Lithgow T., Heinz E., Naderer T. (2018). Outer membrane vesicles from neisseria gonorrhoeae target PorB to mitochondria and induce apoptosis. PLoS. Pathog..

[bib7] Deo P., Chow S.H., Han M.-L., Speir M., Huang C., Schittenhelm R.B., Dhital S., Emery J., Li J., Kile B.T., Vince J.E., Lawlor K.E., Naderer T. (2020). Mitochondrial dysfunction caused by outer membrane vesicles from Gram-negative bacteria activates intrinsic apoptosis and inflammation. Nat. Microbiol..

[bib9] Dos Santos J.A.C., Veras A.S.C., Batista V.R.G., Tavares M.E.A., Correia R.R., Suggett C.B., Teixeira G.R. (2022). Physical exercise and the functions of microRNAs. Life Sci..

[bib10] Ellzey L.M., Patrick K.L., Watson R.O. (2023). Mitochondrial reactive oxygen species: double agents in *Mycobacterium tuberculosis* infection. Curr. Opin. Immunol..

[bib11] Fu D., Li J., Wu J., Shao Y., Song X., Tu J., Qi K. (2021). The ETT2 transcriptional regulator EivF affects the serum resistance and pathogenicity of avian pathogenic *Escherichia coli*. Microb. Pathog..

[bib12] Gao S., Gao L., Yuan D., Lin X., van der Veen S. (2024). Gonococcal OMV-delivered PorB induces epithelial cell mitophagy. Nat. Commun..

[bib13] Groeger S., Meyle J. (2019). Oral mucosal epithelial cells. Front. Immunol..

[bib14] Gupta, S., S. L. Cassel, F. S. Sutterwala, and J. Dagvadorj. Regulation of the NLRP3 inflammasome by autophagy and mitophagy. Available at https://onlinelibrary.wiley.com/doi/10.1111/imr.13410 (verified 16 April 2026). 10.1111/imr.13410 PMC12477832 39417249

[bib15] Han B., Zhou L., Shi Y., Zhao F., Ji J., Zhang K., Yin S., Ning X. (2025). LncRNA432-miR-21-y-DAPK2 ceRNA crosstalk regulates antibacterial response in hypoxia stress through mediating mitochondrial apoptosis in teleost fish. Int. J. Biol. Macromol..

[bib16] He Y., Ding D., Guo J., Sun H., Yang J. (2025). Activation and regulation of NAIP/NLRC4 and NLRP3 inflammasomes during salmonella infection: mechanisms and therapeutic implications. Mol. Biol. Rep..

[bib17] Hu J., Afayibo D.J.A., Zhang B., Zhu H., Yao L., Guo W., Wang X., Wang Z., Wang D., Peng H., Tian M., Qi J., Wang S. (2022). Characteristics, pathogenic mechanism, zoonotic potential, drug resistance, and prevention of avian pathogenic *Escherichia coli* (APEC). Front. Microbiol..

[bib18] Jabir M.S., Hopkins L., Ritchie N.D., Ullah I., Bayes H.K., Li D., Tourlomousis P., Lupton A., Puleston D., Simon A.K., Bryant C., Evans T.J. (2015). Mitochondrial damage contributes to pseudomonas aeruginosa activation of the inflammasome and is downregulated by autophagy. Autophagy.

[bib19] Jiang F., Lou J., Zheng X., Yang X. (2021). LncRNA MIAT regulates autophagy and apoptosis of macrophage infected by *Mycobacterium tuberculosis* through the miR-665/ULK1 signaling axis. Mol. Immunol..

[bib20] Kellermann M., Scharte F., Hensel M. (2021). Manipulation of host cell organelles by intracellular pathogens. Int. J. Mol. Sci..

[bib21] Khairullah A.R., Afnani D.A., Riwu K.H.P., Widodo A., Yanestria S.M., Moses I.B., Effendi M.H., Ramandinianto S.C., Wibowo S., Fauziah I., Kusala M.K.J., Fauzia K.A., Furqoni A.H., Raissa R. (2024). Avian pathogenic escherichia coli: epidemiology, virulence and pathogenesis, diagnosis, pathophysiology, transmission, vaccination, and control. Vet. World.

[bib22] Lenz J.D., Hackett K.T., Dillard J.P. (2017). A single dual-function enzyme controls the production of inflammatory NOD agonist peptidoglycan fragments by neisseria gonorrhoeae. mBio.

[bib23] Li Q., Qi Z., Fu D., Tang B., Song X., Shao Y., Tu J., Qi K. (2023). EspE3 plays a role in the pathogenicity of avian pathogenic escherichia coli. Vet. Res..

[bib24] Li R., Li N., Zhang J., Wang Y., Liu J., Cai Y., Chai T., Wei L. (2016). Expression of immune-related genes of ducks infected with avian pathogenic *Escherichia coli* (APEC). Front. Microbiol..

[bib25] Li Z., Shang D. (2024). NOD1 and NOD2: essential monitoring partners in the innate immune system. Curr. Issues. Mol. Biol..

[bib26] Li Z., Shang W., Mei T., Fu D., Xi F., Shao Y., Song X., Wang Z., Qi K., Tu J. (2024). Outer membrane vesicles of avian pathogenic *Escherichia coli* induce necroptosis and NF-κB activation in chicken macrophages via RIPK1 mediation. Res. Vet. Sci..

[bib27] Lu L., Qi Z., Chen Z., Wang H., Wei X., Zhao B., Wang Z., Shao Y., Tu J., Song X. (2024). Avian pathogenic *Escherichia coli* T6SS effector protein Hcp2a causes mitochondrial dysfunction through interaction with LETM1 protein in DF-1 cells. Poult. Sci..

[bib28] Magaña G., Harvey C., Taggart C.C., Rodgers A.M. (2024). Bacterial outer membrane vesicles: role in pathogenesis and host-cell interactions. Antibiotics.

[bib29] Matsuzawa Y., Oshima S., Takahara M., Maeyashiki C., Nemoto Y., Kobayashi M., Nibe Y., Nozaki K., Nagaishi T., Okamoto R., Tsuchiya K., Nakamura T., Ma A., Watanabe M. (2015). TNFAIP3 promotes survival of CD4 T cells by restricting MTOR and promoting autophagy. Autophagy.

[bib30] Mellata M. (2013). Human and avian extraintestinal pathogenic Escherichia coli: infections, zoonotic risks, and antibiotic resistance trends. Foodborne Pathog. Dis..

[bib31] Mellata M., Dho-Moulin M., Dozois C.M., Curtiss R., Lehoux B., Fairbrother J.M. (2003). Role of avian pathogenic *Escherichia coli* virulence factors in bacterial interaction with chicken heterophils and macrophages. Infect. Immun..

[bib32] Ning X.-H., Han B., Peng Y., Yin S.-W. (2024). LncRNA pol-lnc78 as a ceRNA regulates antibacterial responses via suppression of pol-miR-n199-3p-mediated SARM down-regulation in *Paralichthys olivaceus*. Zool. Res..

[bib33] Peng X., Zhang C., Zhou Z.-M., Wang K., Gao J.-W., Qian Z.-Y., Bao J.-P., Ji H.-Y., Cabral V.L.F., Wu X.-T. (2022). A20 attenuates pyroptosis and apoptosis in nucleus pulposus cells via promoting mitophagy and stabilizing mitochondrial dynamics. Inflamm. Res..

[bib34] Pin C., David L., Oswald E. (2023). Modulation of autophagy and cell death by bacterial outer-membrane vesicles. Toxins.

[bib35] Pokharel P., Dhakal S., Dozois C.M. (2023). The diversity of *Escherichia coli* pathotypes and vaccination strategies against this versatile bacterial pathogen. Microorganisms.

[bib36] Qu Y., Jiang D., Liu M., Wang H., Xu T., Zhou H., Huang M., Shu W., Xu G. (2023). LncRNA DANCR restrained the survival of mycobacterium tuberculosis H37Ra by sponging miR-1301-3p/miR-5194. Front. Microbiol..

[bib37] Reid C.J., McKinnon J., Djordjevic S.P. (2019). Clonal ST131-H22 *Escherichia coli* strains from a healthy pig and a human urinary tract infection carry highly similar resistance and virulence plasmids. Microb. Genom..

[bib38] Ronco T., Stegger M., Olsen R.H., Sekse C., Nordstoga A.B., Pohjanvirta T., Lilje B., Lyhs U., Andersen P.S., Pedersen K. (2017). Spread of avian pathogenic *Escherichia coli* ST117 O78:H4 in nordic broiler production. BMC Genom..

[bib39] Rotimi D.E., Iyobhebhe M., Oluwayemi E.T., Evbuomwan I.O., Asaleye R.M., Ojo O.A., Adeyemi O.S. (2024). Mitophagy and spermatogenesis: role and mechanisms. Biochem. Biophys. Rep..

[bib40] Salmena L., Poliseno L., Tay Y., Kats L., Pandolfi P.P. (2011). A *ceRNA* hypothesis: the rosetta stone of a hidden RNA language?. Cell.

[bib41] Shembade N., Ma A., Harhaj E.W. (2010). Inhibition of NF-κB signaling by A20 through disruption of ubiquitin enzyme complexes. Science.

[bib42] Song Y., Ge X., Chen Y., Hussain T., Liang Z., Dong Y., Wang Y., Tang C., Zhou X. (2022). Mycobacterium bovis induces mitophagy to suppress host xenophagy for its intracellular survival. Autophagy.

[bib43] St Laurent G., Wahlestedt C., Kapranov P. (2015). The Landscape of long noncoding RNA classification. Trends. Genet..

[bib44] Sun D., Gou H., Zhang Y., Li J., Dai C., Shen H., Chen K., Wang Y., Pan P., Zhu T., Xu C., Shan T., Liao M., Zhang J. (2025). Salmonella typhimurium persistently infects host via its effector SseJ-induced PHB2-mediated mitophagy. Autophagy.

[bib45] Sun H., Ma Y., Cao X., Li H., Han W., Qu L.-J., Lamont S.J. (2024). *PTEN* regulated by gga-miR-20a-5p is involved in chicken macrophages inflammatory response to APEC infection via autophagy. Poult. Sci..

[bib46] Tang X., Feng D., Li M., Zhou J., Li X., Zhao D., Hao B., Li D., Ding K. (2019). Transcriptomic analysis of mRNA-lncRNA-miRNA interactions in hepatocellular carcinoma. Sci. Rep..

[bib47] Tiku V., Tan M.-W., Dikic I. (2020). Mitochondrial functions in infection and immunity. Trends. Cell Biol..

[bib48] Wang Z., Zhu D., Zhang Y., Xia F., Zhu J., Dai J., Zhuge X. (2023). Extracellular vesicles produced by avian pathogenic *Escherichia coli* (APEC) activate macrophage proinflammatory response and neutrophil extracellular trap (NET) formation through TLR4 signaling. Microb. Cell Fact..

[bib49] Welsh J.A., Goberdhan D.C.I., O’Driscoll L., Buzas E.I., Blenkiron C., Bussolati B., Cai H., Di Vizio D., Driedonks T.A.P., Erdbrügger U., Falcon-Perez J.M., Fu Q.-L., Hill A.F., Lenassi M., Lim S.K., Mahoney M.G., Mohanty S., Möller A., Nieuwland R., Ochiya T., Sahoo S., Torrecilhas A.C., Zheng L., Zijlstra A., Abuelreich S., Bagabas R., Bergese P., Bridges E.M., Brucale M., Burger D., Carney R.P., Cocucci E., Crescitelli R., Hanser E., Harris A.L., Haughey N.J., Hendrix A., Ivanov A.R., Jovanovic-Talisman T., Kruh-Garcia N.A., Ku’ulei-Lyn Faustino V., Kyburz D., Lässer C., Lennon K.M., Lötvall J., Maddox A.L., Martens-Uzunova E.S., Mizenko R.R., Newman L.A., Ridolfi A., Rohde E., Rojalin T., Rowland A., Saftics A., Sandau U.S., Saugstad J.A., Shekari F., Swift S., Ter-Ovanesyan D., Tosar J.P., Useckaite Z., Valle F., Varga Z., van der Pol E., van Herwijnen M.J.C., Wauben M.H.M., Wehman A.M., Williams S., Zendrini A., Zimmerman A.J., Consortium M., Théry C., Witwer K.W. (2024). Minimal information for studies of extracellular vesicles (MISEV2023): from basic to advanced approaches. J. ExtraCell Vesicl..

[bib50] White J., Suklabaidya S., Vo M.T., Choi Y.B., Harhaj E.W. (2023). Multifaceted roles of TAX1BP1 in autophagy. Autophagy.

[bib51] Xu D., Hu G., Luo J., Cheng J., Wu D., Cheng L., Huang X., Fu S., Liu J. (2023). *Staphylococcus aureus* induces mitophagy to promote its survival within bovine mammary epithelial cells. Vet. Microbiol..

[bib52] Xu T., Xu X., Chu Y., Jiang D., Xu G. (2021). Long-chain non-coding RNA GAS5 promotes cell autophagy by modulating the miR-181c-5p/ATG5 and miR-1192/ATG12 axes. Int. J. Mol. Med..

[bib53] Yang H., Wang Y., Fan H., Liu F., Feng H., Li X., Chu M., Pan E., Teng D., Chen H., Dong J. (2023). Pseudomonas aeruginosa-induced mitochondrial dysfunction inhibits proinflammatory cytokine secretion and enhances cytotoxicity in mouse macrophages in a reactive oxygen species (ROS)-dependent way. J. Zhejiang. Univ. Sci. B.

[bib54] Yu L., Wang H., Zhang X., Xue T. (2025). Two-component system UhpAB facilitates the pathogenicity of avian pathogenic *Escherichia coli* through biofilm formation and stress responses. Avian Pathol..

[bib55] Zhuge X., Sun Y., Jiang M., Wang J., Tang F., Xue F., Ren J., Zhu W., Dai J. (2019). Acetate metabolic requirement of avian pathogenic *Escherichia coli* promotes its intracellular proliferation within macrophage. Vet. Res..

[bib56] Zhuge X., Sun Y., Xue F., Tang F., Ren J., Li D., Wang J., Jiang M., Dai J. (2018). A novel PhoP/PhoQ regulation pathway modulates the survival of extraintestinal pathogenic *escherichia coli* in macrophages. Front. Immunol..

