# Supplementary material for: Outer membrane vesicles secreted by avian pathogenic Escherichia coli promote intracellular survival in macrophages and systemic infection by regulating mitophagy through the MSTRG.11745.1/gga-miR-15b-5p/TNFAIP3 axis
Source: Poult Sci. 2026 Sep 13;105(12):107755. doi: 10.1016/j.psj.2026.107755 (PMC13595299; doi:10.1016/j.psj.2026.107755)
Supplement: Supplementary file 1 [file mmc3.docx]

Table S1. Sequence of gallus MSTRG.11745.1

MSTRG.11745.1 (1681bp)

GATGTTCTGGACAACGTGTGTGGGGTTCTGCAGCATTCCTGTAGTTCACCTGCTCCAGTTGGGAGATTCTTTTGGAGAAGCTCCCCCCTAAGCCTCCTCCTGCCTGAAGGATGCGAGTGTGAAACCTTTGATGATGATTTCCTATGTGCAAGaatgtgtgctgctgctttaagtaagcagcagctgctgttcaAATCCATTAGCAGGTGGAATACCAATTTGCTTACACTGAGATAATTAGTCTCATGAGAAGAGAGTCCTTGCTCTTACTACAAGCCATCTCGCATTGCTGTCCTTCACCTTTCTCCTAGTCCCACAGATTAAATGTCCTCAATGTCTCCAGAAGGTGTATTAAATTCCTGCTTTTTGCTCTTAGAGGTGGATCAAATAGCTTGCTCGTAGACATGAAGCCTGGCTCCCCTCAGTTCTTCTTGCAGAAGTATGAATACCTTGTCTGTACTGAGCTCCATGGGAACATGTAATCTGCTGATACAACTGGAAATGTGAGTTCTGTGGACCTGAACCCACTTTCCAGTATGCTGAGTCAGCCCAGctcaacagcagagcagctttggTTGAAGGAGAGAAATTACTATCCATACAGCAGGCTCCAGTGTAGCTGGCAGGAGCAGGGCCCTCAGGGAGTTAGCATAATATCACAAACATTAATGCTCCCACCTGTATGCAAAGCATTTCCTGTTCTGGTTAGTGACACAAGCTGAGTGTTAGCAGAGCTCCCGTGTTTGCTGCTAAGATGTATCAGCGTGAGGAGAGAGCATCAGTTATCCCAGAGATGAGAAATCCTGGGTTAAAAGCCATTTAAGGTTATAATGTTGGCGTTGGTTTTTCAAGTGAAGTTAGAAGCTACTGAAGTTATCTCATTAGCCACTAATTGCTGAGAGCCTGAATGTAAATTACAGTCATTCCTAGAAGAAGCTGGGTGGCAGTCCCAGCTCCAGGCATGGCTTTGCTTGTGGGTAACAAACAGGATTTTCAAAGGCAAGAATAGAAATGAGACTGCACCCAGAGGAGCCACTGTGTTACTATGCTGATACCGATTTATTCTCTCTAACTGGGACAAGCCTGTGGGATTTATGTCATTCTCTCATACATGCCCTTctgctttggggttttttggtttttttttccttttttagccTTTTTCTTATCCTAAACAATTAAAATGGCTTTTGCTTCAATTATGTGATTCCTAATTTGGCATAAAGGTGGTAGGGCTGGCTGTGGGATTATCAGGCTGTCAGCAGATCAGTACTTTGGGTTCTGACCCCACCAACACAAAGCGGGGTGCAATGAAATAGTTTGAGATTTTATATTGACAATGAGGATTTCATACCATCCAGTAAGATGGTTGCAGATGACTGTGGTCACAGGCAGGATGTGCTTGGTGGTGAGGGCAGCTCTGGTGGACTCATCCAAATGGCAGCTTGGAGGTCTCACATCAGCTGATTGTGAACGTGGAGGAGTCCAACGGTGTTACATCTGCCCTACTTACAGCTGGCTCCGGTGCAGTTCATGGCAAGCTGGCTGCCCTTCTCAAGGGAAAGGTCACTCCAGCATCTGGAGGGAGAAAATCTTGGAGCTATGATTTCAGCCTAGCTGTGGTTCCTCCTTGGGAGGGTTGGCATTATCATCACTAAGACTCTGCACACAGCTTCCACAG
