# Supplementary material for: Outer membrane vesicles secreted by avian pathogenic Escherichia coli promote intracellular survival in macrophages and systemic infection by regulating mitophagy through the MSTRG.11745.1/gga-miR-15b-5p/TNFAIP3 axis
Source: Poult Sci. 2026 Sep 13;105(12):107755. doi: 10.1016/j.psj.2026.107755 (PMC13595299; doi:10.1016/j.psj.2026.107755)
Supplement: Supplementary file 2 [file mmc4.docx]

Table S2. Primer sequences used in this study

| **Primers name** | **Sequence (5ʹ → 3****ʹ)** |
| --- | --- |
| *pKD3-ldcA*-F | ttacattttaagaacaggatgaccgctaatcgtcaactga GTGTAGGCTGGAGCTGCTT |
| *pKD3-ldcA*-R | atgtctctgtttcacttaattgccccntcgggttactgca CATATGAATATCCTCCTTAGTTC |
| *pCP20*-F | TTAGTGGTTGTAAAAACACCTGACC |
| *pCP20*-R | GTTAGCGTTGAAGAATTTAGCCC |
| *ldcA*-F | CTTCCAGCACCAGAATACC |
| *ldcA*-R | GACTGACTACCCCCAACAC |
| *ldcA-out*-F | GTTACTTACCATGTAGGCG |
| *ldcA-out*-R | GTGATCAATTGTGGATCGA |
| *β-actin*-F  *β-actin*-R | CCGCTCTATGAAGGCTACGC  CTCTCGGCTGTGGTGGTGAA |
| *coxII*-F  *coxII*-R  *RPL13A*-F  *RPL13A*-R  *MSTRG.11745.1*-F  *MSTRG.11745.1*-R  *TNFAIP3*-F  *TNFAIP3*-R  *GAPDH*-F  *GAPDH*-R | GCGCAAGTAGGTCTACAAG  GGCAGGATAGTTCAGACGG  CCCCCTACGACAAGAAAAAG  CCGTAGCCTCATGAGCTGTT  GCCTGAAGGATGCGAGTGT  AATGCGAGATGGCTTGTAG  TGGGGCTCGAAACAGACTTC  TGGAGCAGGCCTCTTTTCTG  ATGGTGAAAGTCGGAGTCAACG  TCACTCCTTGGATGCCATGTG |
