# Supplementary material for: Outer membrane vesicles secreted by avian pathogenic Escherichia coli promote intracellular survival in macrophages and systemic infection by regulating mitophagy through the MSTRG.11745.1/gga-miR-15b-5p/TNFAIP3 axis
Source: Poult Sci. 2026 Sep 13;105(12):107755. doi: 10.1016/j.psj.2026.107755 (PMC13595299; doi:10.1016/j.psj.2026.107755)
Supplement: Supplementary file 3 [file mmc5.docx]

Table S3. Sequences of miRNA mimics and siRNAs used in this study

| **Target gene/miRNA** | **Reagent type** | **Sequence (5′** **→ 3′)** |
| --- | --- | --- |
| gga-miR-15b-5p mimics | miRNA mimic | UAGCAGCACAUCAUGGUUUGCA |
| si-*TNFAIP3*  si-MSTRG.11745.1 | siRNA  siRNA | GACAGCUUGCUCCUUAUAA  GUGCAAGAAUGUGUGCUGCUG |
